# A nanoparticle vaccine that targets neoantigen peptides to lymphoid tissues elicits robust antitumor T cell responses

**DOI:** 10.1038/s41541-020-00253-9

**Published:** 2020-11-12

**Authors:** Carlos A. Arbelaez, Juan Estrada, Melissa A. Gessner, Charles Glaus, Agnieszka B. Morales, Deanna Mohn, Hyewon Phee, J. Russell Lipford, James A. Johnston

**Affiliations:** 1 Department of Inflammation and Oncology, Amgen Research, Amgen Inc, One Amgen Center Drive, Thousand Oaks, CA 91320 USA; 2 Department of Clinical Immunology, Translational Medicine, Amgen Inc, One Amgen Center Drive, Thousand Oaks, CA 91320 USA; 3 Department of Research Imaging Sciences, Amgen Research, Amgen Inc, One Amgen Center Drive, Thousand Oaks, CA 91320 USA; 4 Department of Inflammation and Oncology, Amgen Research, Amgen Inc, 1120 Veterans Blvd, South San Francisco, CA 94080 USA

**Keywords:** Peptide vaccines, Cancer immunotherapy

## Abstract

Cancer vaccines using synthetic long peptides (SLP) targeting tumor antigens have been tested in the clinic but the outcomes have been unimpressive, perhaps because these peptides elicit predominantly CD4^+^ T cell responses. We hypothesized that enhanced delivery of peptide antigens to, and uptake in, secondary lymphoid tissues should elicit more robust CD8^+^ and CD4^+^ T cell responses and improved anti-tumor responses. Here, we have designed SLP-containing cationic lipoplexes (SLP–Lpx) that improve delivery of peptides to myeloid cells in the spleen and lymphatics. Using the G12D KRAS mutations as neoantigens, we found that vaccination of mice with naked synthetic peptides harboring the G12D mutation with CpG adjuvant stimulated mainly CD4^+^ T cell responses with limited tumor growth inhibition. On the other hand, immunization with SLP–Lpx stimulated both CD4^+^ and CD8^+^ T cells and suppressed tumor growth in a CD8^+^ T cell-dependent manner. Combination of the SLP–Lpx vaccines with a checkpoint inhibitor led to profound growth suppression of established tumors. These studies suggest that preferential targeting of peptides derived from neoantigens to the spleen via lipoplexes elicits potent CD4^+^ and CD8^+^ T cell responses that inhibit tumor growth.

## Introduction

Neoepitopes, arising from tumor-specific mutations and presented on major histocompatibility (MHC) class I and/or II, are unique to the tumor and represent “non-self”, making them attractive targets for tumor vaccine therapy. Despite the challenge of reliably predicting immunogenic peptide-MHC neoantigens, current prediction methods for peptide binding to common MHC-I alleles have greatly improved. As such, the next challenge is to discover a potent vaccination platform that stimulates sufficient tumor antigen-specific effector T cells to inhibit tumor progression.

Vaccination strategies have focused on delivering potential antigens to antigen-presenting cells (APCs), mainly dendritic cells (DCs). While exogenous peptide antigens are taken up via the endocytic pathway and presented on MHC class II on DCs, MHC class I molecules are loaded with endogenous antigens in the cytosol. However, specialized DCs, such as CD8a^+^ DCs, have the ability to cross-present exogenous antigens to CD8^+^ cytotoxic T lymphocytes (CTLs) to initiate immune responses against tumors^1^. Therefore, efficient delivery of neoantigen peptides to DCs should provide enhanced CTL responses and more effective of immunotherapy^2^.

Although safe, peptide vaccines' success has been limited due to poor targeting, reduced accumulation in draining lymph nodes, and low immunogenicity^3^. This is partly due to failed -accumulation of the antigens (peptides) to the draining lymph nodes, where local immune responses could be initiated by cross-presenting DCs^3^. Furthermore, exogenous peptide uptake into DCs preferentially activates CD4^+^ T cells due to limited peptide access to the cytosol and proteasome, characteristics required for cross-presentation^4^. Recent clinical studies of neoantigen cancer vaccines report similar observations, in which the peptide neoantigen cancer vaccines predominantly induced CD4^+^ T cells despite the peptides being selected based on MHC class I predictions^5,6^. Although the reason CD4^+^ T cell responses were dominant is not well-understood, peptide delivery to the draining lymph nodes or spleen, where antigens can be taken up by resident DCs, should facilitate robust CD8^+^ T cell responses. In addition, targeting neoantigen peptides to the secondary lymphoid organs should also activate CD4^+^ T cells^7–10^. Of importance, activating CD4^+^ T cells in addition to neoantigen-specific CTLs leads to enhanced anti-tumor responses. Recently, Shreiber et al. described a key concept for the generation of vaccines: efficacious vaccines require MHC-I and MHC-II antigenic epitopes that reside within the same peptide region, resulting in a more efficient presentation by the same APCs^11^. This study identified an important role for CD4^+^ T cell-specific antigens in the tumor microenvironment, even in tumors that did not express MHC class II. CD4^+^ T cell help was required for optimal CTL generation that killed tumor cells^11^. Furthermore, interaction with CD4^+^ T cells stimulates DCs to produce cytokines, such as IL-15, and upregulate CD80/86 and CD70, enabling potent CD8^+^ T cell responses^12^. Thus, vaccine platforms that induce both CD4^+^ and CD8^+^ T cell responses would be highly desirable to enhance the therapeutic efficacy of a neoantigen-directed cancer vaccine.

In this study, we describe a peptide-based cancer vaccine platform that elicits both CD4^+^ and CD8^+^ T cell responses toward neoantigens. The G12 mutation of KRAS is one of the main driver mutations in human cancer, which is now thought to be presented on some pMHC alleles in some patients^13,14^. Due to the relative safety of synthetic long peptides (SLPs) and cationic liposomes in the clinic, we designed SLP-containing cationic lipoplexes (SLP–Lpx) to deliver neoantigen peptides to myeloid cells in the spleen, including CD8^+^ DCs^15^. This liposome-based peptide delivery platform induced both CD4^+^ and CD8^+^ T cell responses to KRAS G12 pMHC epitopes, unlike peptide vaccination that mainly induced CD4^+^ T cell responses. When coupled with checkpoint blockade, the SLP–Lpx vaccine efficiently inhibited the growth of established tumors. These data indicate that this peptide lipoplex approach can induce both CD4^+^ and CD8^+^ T cell responses to neoantigens to efficiently control tumor growth.

## Results

### Immunization of naive mice with mutant KRAS peptides identifies several T cell epitopes and generates mainly CD4^+^ T cell responses

**Fig. 1:**
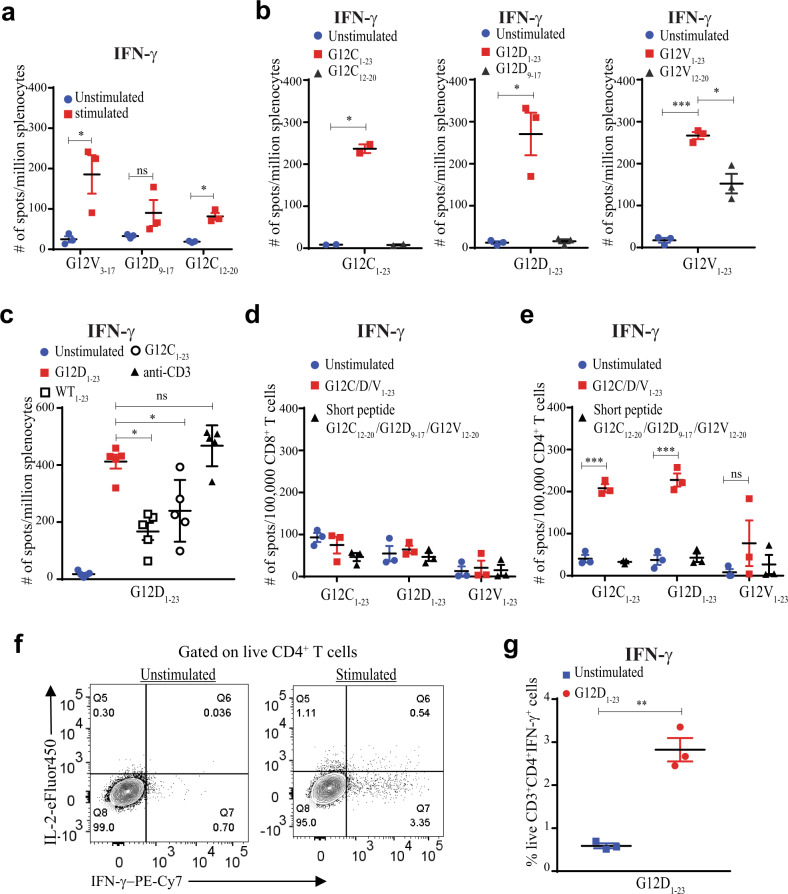
Peptides containing the KRAS G12C, G12D, and G12V peptides are immunogenic in mice and stimulate mainly CD4^+^ T cell responses. **a** C57Bl/6 mice were immunized with indicated peptides on days 0 and 7. Reactivity was determined by ELISpot using splenocytes re-stimulated on day 14. **b** Mice immunized as in **a** with G12C_1–23_, G12D_1–23_, or G12V_1–23_ and re-stimulated with the immunizing peptide or 9 or 15-mer. **c** Splenocytes from G12D_1–23_-immunized mice were re-stimulated with WT or G12C 23-mer peptides. **d** IFN-γ ELISpot results after co-culture of BMDCs pulsed with the immunizing peptide with CD8^+^ T cells from the spleens of G12C_1–23_-, G12D_1–23_-, and G12V_1–23_-immunized mice. **e** Peptide-specific IFN-γ secretion from CD4^+^ T cells co-cultured with peptide-pulsed BMDCs. **f**, **g** G12D_1–23_ peptide-specific CD4^+^ T cells measured by intracellular cytokine staining by cytometry. Significance determined using two-way ANOVA (**a**, **c**) and paired two-tailed Student’s *t*-test (***p < 0.001, **p < 0.01, *p < 0.05. Error bar = mean ± s.e.m.) (**a-e**, **g**). Results from at least two independent studies with two to five mice each.

The KRAS G12 variants are among the most frequent mutations found in human gastrointestinal and lung cancers. Peptides derived from these recurrent KRAS mutations (G12D/C/V) have the potential to bind several HLA class I alleles with relatively high affinity, as shown by in silico prediction (Supplementary Fig. 1a, b). Previous studies suggest that these may also be presented on rarer class I alleles^13^. Although there have been reports of KRAS mutations stimulating T cell responses in mice^16^, few studies have explored the use of longer peptides that may potentially include several epitopes for CD4^+^ and CD8^+^ T cells. To explore whether MHC class I-restricted CD8^+^ T cell responses can be generated by KRAS G12 variants, we performed IFN-γ ELISpot screens of individual, overlapping 9-mer peptides in C57Bl/6 mice after immunization with peptide and CpG (Supplementary Fig. 1c–e). Only peptides G12C_12–20_ and G12D_9–17_ elicited responses after immunization and re-stimulation (Fig. 1a). However, the response to the G12C_12–20_ peptide was consistently less robust. The 9-mer peptides containing the G12V mutation did not elicit an IFN-γ ELISpot response, but the 15-mer containing the G12V mutation (G12V_3–17_) strongly stimulated T cell responses (Fig. 1a), as previously reported^16^. To identify other potentially immunogenic epitopes, we used 23-mer peptides from KRAS bearing each of the mutations: G12C peptide (G12C_1–23_), G12D (G12D_1–23_), and G12V (G12V_1–23_) with mutations at residue 12. Mice immunized with CpG alone were used as controls. The 23-mer mutated peptides all stimulated potent T cell responses (Fig. 1b) but did not elicit any class I-restricted CD8^+^ T cell responses since the re-stimulation of splenocytes from these immunized mice with the immunogenic 9-mer peptides G12C_12–20_ or G12D_9–17_ did not stimulate IFN-γ production. (Fig. 1b). It is likely that class I responses are undetectable due to the predominant processing of longer peptide epitopes through the endocytic pathway, preventing class I peptide loading in the endoplasmic reticulum. In line with this idea, stimulation with the immunogenic G12V 15-mer resulted in about half as many IFN-γ spots as with the immunizing 23-mer (Fig. 1b), suggesting a predominant presentation of longer peptides by MHC class II molecules.

T cells generated by vaccinating with G12D_1–23_ peptide produced IFN-γ following re-stimulation with WT_1–23_ or G12C_1–23_ peptides, albeit with less potency compared to G12D_1–23_ re-stimulation, suggesting some T cells generated by this vaccination approach recognized WT_1–23_ or G12C_1–23_ peptides (Fig. 1c). These results demonstrate that vaccination with SLP harboring G12 KRAS mutations induce T cell responses against mutant KRAS peptides.

To determine which T cell subtypes were being stimulated by SLP vaccination, we isolated CD4^+^ and CD8^+^ T cells from mice immunized with the SLPs. Twelve days after the first vaccination with G12C_1–23_, D_1–23_, or V_1–23_ peptides with CpG, splenocytes were re-stimulated with peptide-pulsed bone marrow-derived dendritic cells (BMDCs). We found no antigen-specific CD8^+^ T cells after re-stimulation with the immunizing SLP or the short peptides found to elicit IFN-γ in Fig. 1a (Fig. 1d). However, the G12C/D/V_1–23_ peptides elicited CD4^+^ T cell responses (Fig. 1e). The antigen-specific CD4^+^ T cells from the G12D_1–23_-immunized mice produced high levels of IFN-γ and could be cultured in vitro for at least 12 days (Fig. 1f,g).

Overlapping 15-mer peptides spanning the entirety of the G12D_1–23_ 23-mer were pooled to match the immunizing peptide (Supplementary Fig 2a). When restimulating with three pools of overlapping 15-mers, we found that 2 peptides (G12D_9–17_ and G12D_8–16_) consistently elicited CD4^+^ T cell responses in an IFN-γ ELISpot assay (Supplementary Fig. 2b), while WT peptides did not (Supplementary Fig. 2c). These results suggest the generation of potent CD4^+^ T cell responses against the KRAS-G12D mutation.

### Peptide lipoplexes boost immunogenicity to CD8^+^ T cell epitopes

**Fig. 2:**
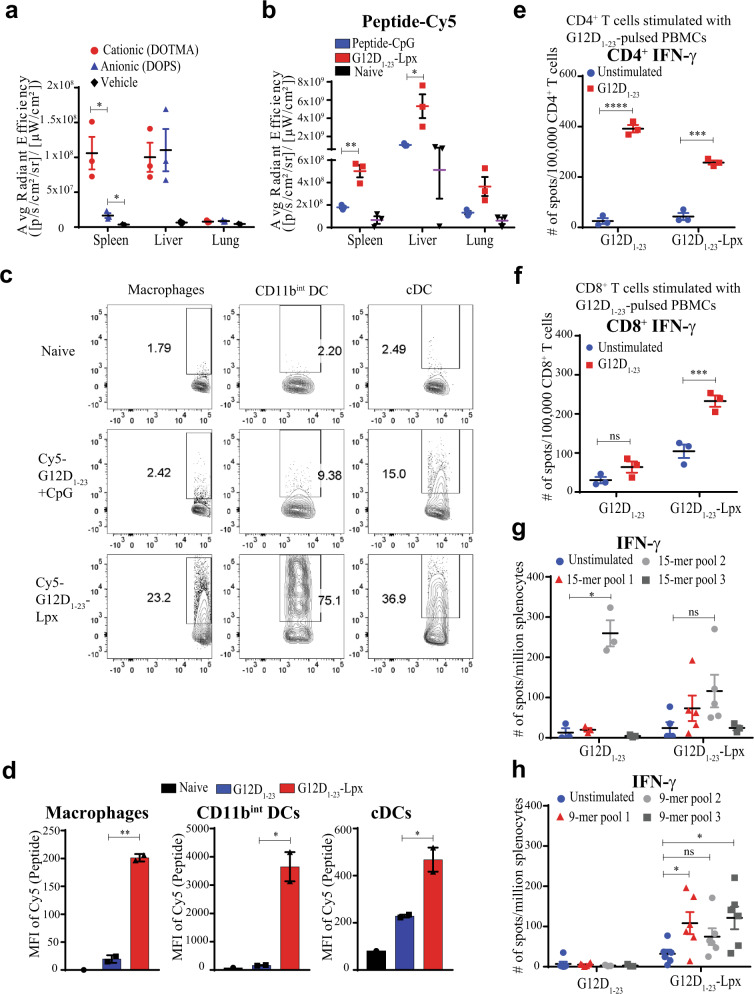
Peptide formulation into liposomes boosts immunogenicity and improves processing and presentation. **a** Biodistribution of rhodamine-labeled cationic and anionic liposomes delivered i.v. 30 min before harvest and assessment by IVIS. **b** Cy5-labeled G12D_1–23_ peptide delivered i.v. with CpG or incorporated into cationic liposomes (Lpx). Spleen, liver, and lung biodistribution assessed by signal intensity. **c**, **d** Cy5-labeled G12D_1–23_ peptide uptake by CD11b^+^CD11c^−^ macrophages, CD11b^+^ DCs, and CD11b^int^CD11c^+^ cDCs 30 min after s.c. immunization of mice with Cy5-labeled G12D_1–23_ peptide and CpG or G12D_1–23_ peptide–liposome–CpG (G12D_1–23_–Lpx). **e**, **f** Comparison of G12D_1–23_-specific CD4^+^ and CD8^+^ T cell responses after co-culture of peptide-pulsed BMDCs with T cells isolated from peptide- or peptide–Lpx-immunized mice. **g**, **h** Mice were immunized with naked G12D_1–23_ peptide or peptide–Lpx and splenocytes were re-stimulated with pools of overlapping 9- or 15-mers. IFN-γ responses were determined by ELISpot. Significance determined using two-way ANOVA (**a**, **b**, **g**, **h**) and one-sided or paired two-tailed Student’s *t*-test (***p < 0.001, **p < 0.01, *p < 0.05. Error bar = mean ± s.e.m.).

Peptide immunization is attractive due to safety profiles and ease of manufacturing. However, one of the shortcomings of current peptide immunization strategies is the poor lymphatic biodistribution and resultant limited immunogenicity. A recent study illustrated the successful use of negatively charged mRNA-containing cationic liposomes to induce potent antigen-specific responses^17^. Using rhodamine-labeled lipid DOPE as a helper lipid, we confirmed that empty cationic liposomes biodistribute to the spleen better than anionic liposomes when administered intravenously. These data argue that to deliver antigens to the spleen, the composition of the liposome is more critical than the overall charge of the liposome (Fig. 2a). If liposome charge plays a negligible role, then one could mix the lipid with peptides and generate peptide–liposome complexes (SLP–Lpx) with similar biodistribution profiles. Delivery of peptide antigens to the spleen and lymph nodes, particularly in lipoplexes, should facilitate uptake and presentation by different dendritic cell (DC) subtypes and facilitate cross-presentation. Thus, we hypothesized that liposome-mediated delivery of SLP should target the peptide antigens to the spleen and lymph nodes, facilitating both CD4^+^ and CD8^+^ T cell responses.

To determine the appropriate liposome composition to facilitate optimal lymphatic targeting, we tested several methods and formulations using subcutaneous immunization to generate the KRAS peptide G12D_1–23_–liposome complex using IFN-γ release after SLP re-stimulation in the spleen as a readout. We determined that incubation and extrusion of 5 μg of the peptide with 50 μg of liposomes with cationic lipids consistently resulted in stable 100–150 nm liposomes (Supplementary Fig 3a), a positive zeta potential, and the strongest generation of G12D-_1–23_-specific T cell responses (Supplementary Fig. 3b). CpG was added to the peptide–liposome to make peptide–liposome–CpG (henceforth termed G12D_1–23_–Lpx) to enhance the adjuvant effect. When comparing anionic and cationic peptide–Lpx, we confirmed that subcutaneous administration of cationic liposomes elicits more robust splenic targeting (Supplementary Fig. 3c). Thus, we selected the cationic liposome formulation to complex with G12D_1–23_ for further analysis.

Subcutaneous administration of Cy5-labeled peptide–lipoplex revealed a 2–3-fold increase in peptide distribution to the spleen when the peptide was complexed to liposomes containing CpG (G12D_1–23_–Lpx), compared to uncomplexed naked Cy5-labeled G12D_1–23_ peptide with CpG (Fig. 2b). Moreover, the percentages of splenic myeloid cells, specifically CD11b^+^CD11c^−^ macrophages, CD11b^+^ dendritic cells (DCs), and CD11b^−^CD11c^+^ classical dendritic cells (cDCs), that took up the Cy5-labeled peptide were dramatically increased when peptides were administered using the peptide–Lpx platform (Fig. 2c and Supplementary Fig. 4). The mean fluorescent intensity (MFI) of the Cy5-labeled peptide taken up by splenic macrophages, CD11b^+^ DCs, and cDCs was increased up to 100-fold when delivered in lipoplexes compared with naked peptide (Fig. 2d).

Increased targeting of the peptides using peptide–Lpx to myeloid cells consistently elevated antigen-specific CD8^+^ T cell responses while maintaining CD4^+^ T cell responses comparable to peptide immunization (Fig. 2e, f). Strong CD8^+^ T cell responses toward MHC class I-restricted epitopes are likely to be driven by macropinocytosis of the peptide–Lpx by DC since the macropinocytosis inhibitors rottlerin and cytochalasin D, which inhibits both macropinocytosis and phagocytosis, both blocked uptake (Supplementary Fig. 5a, b). Macropinocytosis should enable antigens to access the cytosol and be presented on MHC class I^18^, as well as MHC class II by the lysosomal-mediated mechanism.

We postulated that the enhanced myeloid uptake of SLP–Lpx by macropinocytosis might enable the presentation of a larger set of peptide epitopes to both CD4^+^ and CD8^+^ T cells. Indeed, the numbers of overlapping 15-mer peptides activating CD4^+^ T cells were increased when G12D_1–23_–Lpx was used for vaccination (Fig. 2g**)**. While CD8^+^ T cells did not respond when mice were immunized with naked peptide, CD8^+^ T cells recognizing multiple G12D KRAS 9-mer peptides were induced when the G12D_1–23_–Lpx was used for vaccination (Fig. 2h). These results suggest that strong CD4^+^ and CD8^+^ T cell responses were generated towards SLPs harboring CD4^+^ and CD8^+^ T cell epitopes when targeted to the spleen using the lipoplex formulation.

### A single antigen liposome-based vaccine targeting mutant KRAS mediates reduced tumor burden in a GEM model driven by KRAS-G12D

To explore whether enhancing CD8^+^ T cell responses to mutant KRAS inhibits tumor growth, we used an inducible lung adenocarcinoma model in which expression of oncogenic KRAS-G12D is induced after intra-tracheal delivery of Cre expressing adenovirus, Adeno-Cre^19^. Tumor development is accelerated in the absence of LKB1^20^ and insertion of a ROSA26-lox-STOP-lox allele of firefly luciferase marks the cells expressing KRAS and with LKB1 deleted^21^. A vaccine containing either empty Lpx control or G12D_1–23_–Lpx was given one week after Adeno-Cre administration and boosts were administered weekly for 3 weeks. Treating tumors with G12D_1–23_–Lpx greatly reduced tumor volume (Supplementary Fig. 6a–c). Consistent with the reduction in tumor size, we found increased numbers of hematopoietic and T cell infiltrates (Supplementary Fig. 6d, e) However, we did not see differences in Foxp3 or PD-1 expression in the tumors from mice treated with the Lpx control or the G12D_1–23_–Lpx (Supplementary Fig. 6f, g). The lung-infiltrating T cells were reactive to the immunizing peptide, but not to the WT KRAS peptide (Supplementary Fig. 6h). These tumors responded well to the vaccine and ~20% of the mice had few detectable tumors (Supplementary Fig. 6a–c). Interestingly and consistent with previous results, depletion of regulatory T cells with anti-CD25 led to a significant reduction in tumor burden (Supplementary Fig 6a–c), but with reduced infiltrate. These results indicate that a vaccine solely targeting mutant KRAS can inhibit the of KRAS-driven lung adenocarcinoma growth in mice.

### Delivering neoantigen peptides in lipoplexes reduces tumor burden in syngeneic tumor models expressing KRAS mutations

**Fig. 3:**
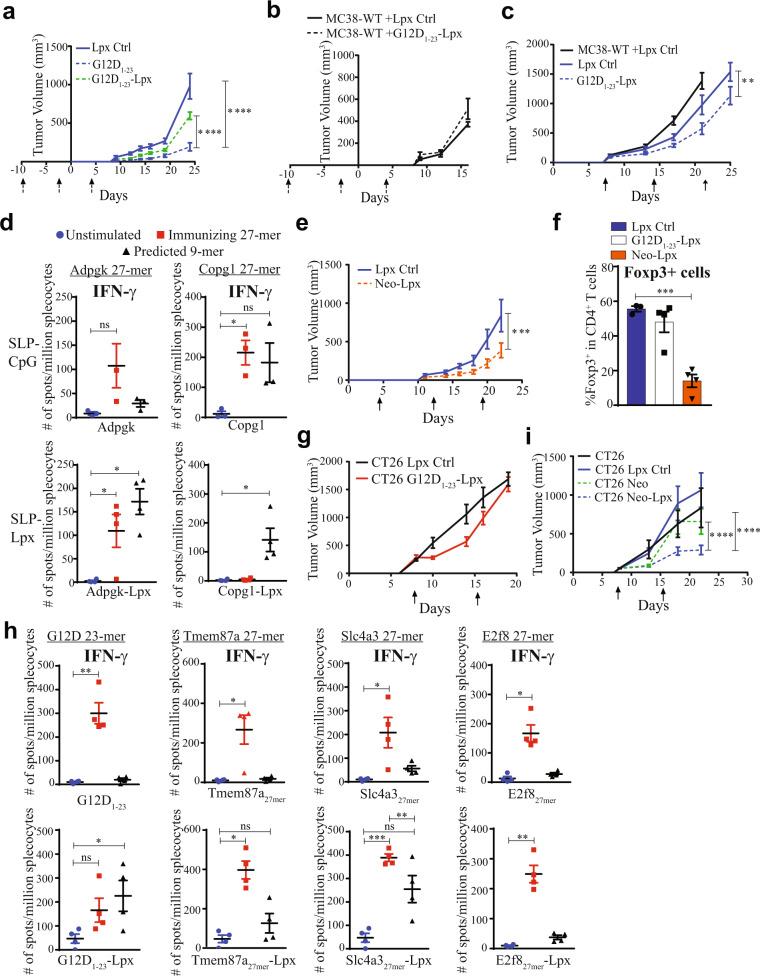
Vaccination with neoantigen peptide–lipoplexes enhances CD8^+^ T cell responses and controls tumor growth in syngeneic tumors expressing mutant KRAS. **a** Growth of MC38-G12D tumors in C57Bl/6 mice immunized with empty liposomes and CpG control (+Lpx Ctrl), G12D_1–23_ peptide and CpG (+G12D_1–23_), or with G12D_1–23_ in lipoplexes (+G12D_1–23_–Lpx) starting 10 days before implantation and every 7 days for 3 weeks (dashed arrows) (n = 5–10). **b** Growth of MC38-WT tumors in mice immunized with G12D_1–23_–Lpx as indicated by the dashed arrows (*n* = 5). **c** Growth of MC38-WT and MC38-G12D tumors in mice immunized with G12D_1–23_–Lpx starting at randomization on days 8 and 7 days later (arrows) (n = 7–9). **d** Mice were immunized with the mutant Adpgk and Copg1 27-mer peptides and CpG (top) and with the 27-mer complexed Lpx twice, one week apart. Splenocytes were stimulated with the immunizing peptide and the 9-mer that stimulates CD8^+^ T cells (*n* = 3). **e** Growth curves of mice immunized with the Adpgk, Copg1, and G12D_1–23_ SLP–Lpx (Neo-Lpx) at the indicated time points (arrows) (*n* = 9–11). **f** Tumor-infiltrating Foxp3^+^CD4^+^ regulatory T cells were quantified by intranuclear staining on day 23. **g** CT26 tumor growth in mice immunized with empty Lpx control or G12D_1–23_–Lpx on the day of randomization and 1 week later (*n* = 9–10). **h** Naive Balb/c mice were s.c. immunized with the indicated neoantigen peptides in the naked form (top) or with Lpx (bottom) once weekly for 2 weeks and an IFN-γ ELISpot was performed to detect reactivity to the immunizing SLP or 9-mer corresponding to the predicted CD8^+^ T cell epitope. **i** Randomized CT26 tumors were immunized with empty Lpx control, CpG, and neoantigen (Neo) peptides (KRAS-G12D_1–23_, Tmem87a G63R 27-mer, and Slc4a3 T373I 27-mer), or neoantigen peptide–lipoplexes (Neo-Lpx). Mice immunized twice after randomization (*n* = 7–11). Significance was determined using two-way ANOVA and one-sided Student’s *t*-test (****p < 0.0001, ***p < 0.001, *p < 0.05. Error bar = mean ± s.e.m.).

We next explored the effect of the SLP-lipoplex vaccine in two syngeneic tumor models in C57Bl/6 and Balb/c mice. We expressed the wild-type (WT) KRAS or the G12D mutation in MC38 cells, which grew slightly faster than the parental MC38 cell line (Supplementary Fig. 7). We tested the SLP–Lpx vaccine in prophylactic and therapeutic settings. Vaccination against KRAS-G12D with the SLP–Lpx before implantation of the MC38-G12D tumors markedly inhibited tumor growth (Fig. 3a, b). The KRAS-G12D_1–23_ SLP–Lpx vaccine activating both CD4^+^ and CD8^+^ T cells was superior to vaccination with the naked peptide and CpG (Fig. 3a). Of note, KRAS WT SLP–Lpx did not impact tumor growth (Fig. 3b).

Because we saw tumor control from G12D_1–23_–Lpx when vaccinated prophylactically but not in WT-Lpx nor in naked peptide-CpG, we performed an efficacy study of the Lpx vaccine in a therapeutic setting. Eight days after tumor implantation, mice were randomized and vaccinated with the KRAS mutant SLP–Lpx vaccine (G12D_1–23_–Lpx) and boosted after 1 week. Tumor growth was consistently inhibited by both vaccines (Fig. 3c). Although the growth rate was markedly reduced, the tumors continued to grow. This may be due to T cell exhaustion induced by vaccination by upregulating checkpoint molecules, such as PD-1, or neoantigen editing that allow cancer cells to adapt to the driver mutations such as KRAS G12 mutations^13^. To determine whether other peptide neoantigens delivered in lipoplexes can also boost CD8^+^ T cell responses, we used a previously reported neoantigen in the C57Bl/6 MC38 model (Adpgk)^22^ and Copg1, which we discovered was mutated in MC38 cells and has a high affinity for MHC Class I (Supplementary Table 1). We immunized C57Bl/6 mice with the mutant 27-mer Adpgk and Copg1 peptides and re-stimulated with either the immunizing 27-mer peptides or 9-mers predicted and demonstrated to be immunogenic. Immunization with either Adpgk or Copg1 SLP with CpG generated T cell responses that could be re-stimulated with the immunizing 27-mer. Interestingly for Adpgk, no responses were detected when re-stimulated with the 9-mer mutant peptide, suggesting that the CD4^+^ T cell responses were dominant and CD8^+^ T cells recognizing 9-mer epitopes were poorly induced (Fig. 3d). However, when the same Adpgk SLP was delivered in a lipoplex platform, both CD4^+^ and CD8^+^ T cell responses were observed (Fig. 3d). Interestingly, immunization with the Copg1 27-mer SLP-induced CD8^+^ T cell responses together with CD4^+^ T cells. However, vaccination with mutant Copg1 SLP–Lpx strongly induced CD8^+^ and not CD4^+^ responses, highlighting the propensity of the SLP–Lpx vaccination to induce CD8^+^ T cell responses (Fig. 3d).

Using the MC38 model with forced expression of KRAS-G12D, we combined the 27-mers encoding Adpgk and Copg1 neoantigens with the KRAS-G12D 23-mer in lipoplexes, termed Neo-Lpx. The first vaccine priming was administered on day 5 followed by a boost at day 12. Indeed, upon vaccination of mice bearing MC38 tumors with Neo-Lpx, we observed a clear and significant delay in tumor growth in the vaccinated mice (Fig. 3e). These responses were comparable to the G12D_1–23_–Lpx vaccination in that there was a delay in tumor growth without complete responses. Interestingly, immunization with the Neo-Lpx led to a profound decrease in the frequencies of Foxp3^+^CD4^+^ regulatory T cells in the tumors (Fig. 3f). These results highlight the increase in T cell effector functions when we immunize with multiple SLP–Lpx.

We used the CT26 model in Balb/c mice (CT26 (*KRAS p.G12D*)) since KRAS-G12D is expressed at lower endogenous levels. Using this approach, we examined whether T cells induced by vaccination could respond to the KRAS mutations and ultimately inhibit tumor growth. However, in this CT26 model, no effect of therapeutic vaccination with the G12D_1–23_–Lpx was observed (Fig. 3g), perhaps due to lower levels of KRAS-G12D expression, preventing adequate peptide presentation.

To explore this model further, we examined the effect of vaccinating against multiple previously reported CT26-specific neoantigens^23^, and used the KRAS-G12D_1–23_ peptide in conjunction with several 27-mers. In naive mice immunized with the peptides Tmem87a, Slc4a3, and E2f8 either with CpG or in lipoplexes, we observed a consistent pattern of SLP–Lpx-specific activation of CD8^+^ T cells using putative neoantigens derived from the CT26 model described previously^23^. Out of four peptides tested that generated responses, three (KRAS-G12D, Tmem87a, and Slc4a3) generated a CD8^+^ T cell response against the tested 9-mer when the peptide was delivered in a lipoplex (Fig. 3h), highlighting the propensity of the SLP–Lpx vaccination to induce CD8^+^ T cell responses. We then immunized CT26 tumor-bearing mice with these three neoantigens in lipoplex form, termed Neo-Lpx. Comparing CT26 tumors in mice immunized with the Neo-Lpx on day 8 after randomization, we observed that immunizing with neoantigen-derived peptides delivered in lipoplexes suppressed tumor growth more profoundly than with naked peptides (Fig. 3i). These results are summarized in Supplementary Table 1.

### Tumor growth inhibition from peptide–lipoplex vaccination is dependent on peptide-specific effector CD8^+^ T cells

**Fig. 4:**
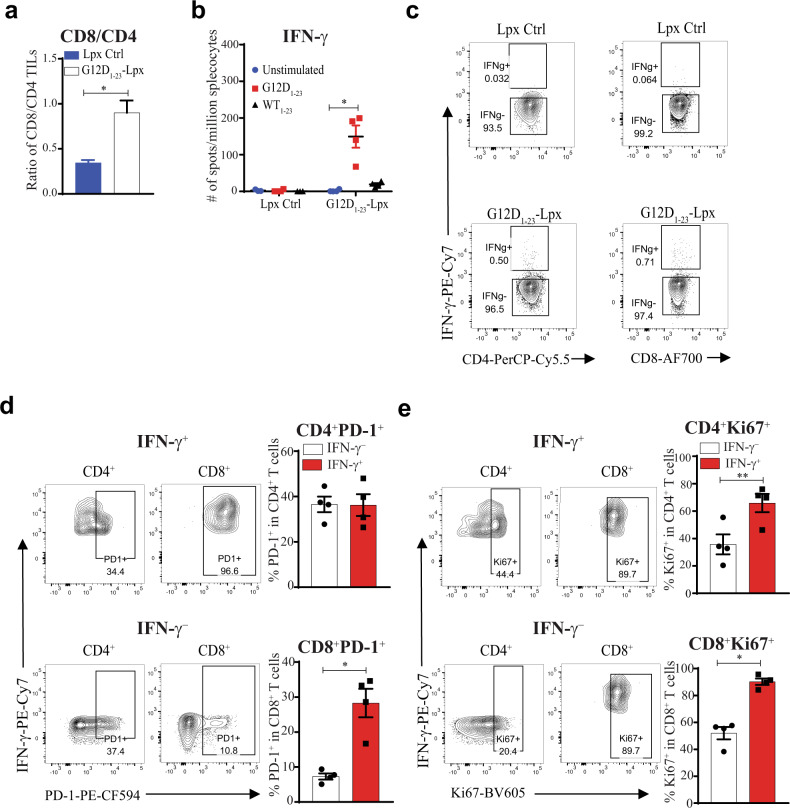
Vaccination with neoantigen peptide–Lpx generates peptide-specific effector CD4^+^ and CD8^+^ T cells. **a** Tumors and spleens from MC38-G12D tumor-bearing mice that were immunized with the G12D_1-23_-Lpx were analyzed on day 25. Analysis of CD8^+^ and CD4^+^ T cell infiltrating the tumors determined by the CD8/CD4 ratio. **b** IFN-γ ELISpot of splenocytes re-stimulated with the immunizing G12D_1–23_ or WT peptide in tumor-bearing mice. **c** Intracellular cytokine staining of CD4^+^ and CD8^+^ T cells from spleens of MC38-G12D tumor-bearing mice after G12D_1–23_ peptide re-stimulation**. d**, **e** Expression of PD-1 and Ki67 based on IFN-γ production from G12D_1–23_-stimulated CD4^+^ and CD8^+^ T cells gated from **c**. (**p < 0.01, *p < 0.05. Error bar = mean ± s.e.m. *n* = 4).

We analyzed the T cell infiltrate in the MC38-G12D model 25 days after tumor transplantation. An increase in the CD8/CD4 tumor-infiltrating T-lymphocyte (TIL) ratio was observed in the peptide–lipoplex-treated group (Fig. 4a) and T cells in the spleen were KRAS-G12D-specific and did not respond to WT KRAS peptide (Fig. 4b). Both KRAS-G12D-specific CD4^+^ and CD8^+^ T cells produced IFN-γ and showed high rates of proliferation, as measured by Ki67, but the antigen-specific T cells had higher levels of PD-1 expression (Fig. 4c-e). These findings argue that enhancing anti-tumor responses with a lipoplex vaccine that stimulates both CD4^+^ and CD8^+^ T cells against a single driver mutation can markedly slow tumor growth by recruiting more CD8^+^ TILs.

**Fig. 5:**
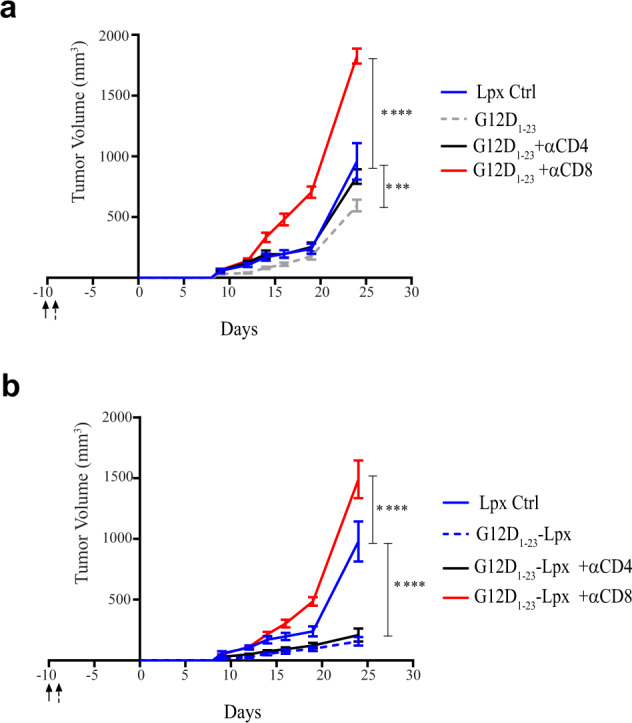
The impact of KRAS peptide–Lpx on tumor growth was completely dependent on CD8^+^ T cells while depletion of CD4^+^ T cells had no impact. **a**, **b** Depletion of CD4^+^ or CD8^+^ T cells before and during the growth of MC38-G12D tumors (solid arrow; every 2–3 days) in mice immunized with the naked peptide G12D_1–23_ and CpG (**a**) or with G12D_1–23_–Lpx starting on day −8 (dashed arrow) (**b**). Significance was determined using two-way ANOVA (****p < 0.0001, ***p < 0.001, *p < 0.05. Error bar = mean ± s.e.m. *n* = 10).

We depleted either CD4^+^ or CD8^+^ T cells using ADCC to determine which T cell subset was predominantly responsible for the effect of the peptide–lipoplex vaccine. Depletion of either CD4^+^ or CD8^+^ T cells before vaccination and throughout the tumor study allowed us to dissect which T cell subset is responsible for the observed effect of the peptide and SLP–Lpx vaccines. The observations suggest that while CD4^+^ T cell depletion had a minor impact, CD8^+^ T cell depletion resulted in markedly enhanced tumor growth, showing, as expected, that CD8^+^ T cells provide endogenous tumor control (Fig. 5a). Moreover, the G12D_1–23_–Lpx vaccine effect was completely dependent on CD8^+^ T cells and depletion of CD4^+^ T cells had little impact on tumor growth (Fig. 5b) These findings support our previous observations that lipoplexes can elicit MHC class I-restricted responses that are not detected when the peptide is administered alone.

**Fig. 6:**
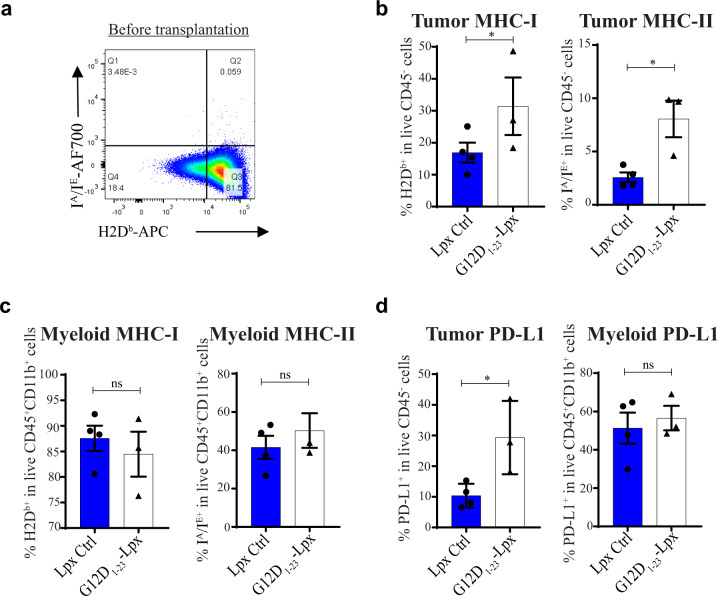
Tumors respond to the peptide–lipoplex vaccine by upregulating MHC class I and II molecules and PD-L1. **a** MHC class I (H2D^b^) expression on MC38-G12D cells before tumor implantation. **b**, **c** MHC class I and MHC class II expression on MC38-G12D tumors or CD11b^+^ myeloid cells from mice immunized with lipoplex (Lpx) control or G12D_1–23_ peptide–lipoplex. **d** PD-L1 expression analyzed by flow cytometric analysis on MC38-G12D tumors and tumor-infiltrating myeloid cells in mice immunized with Lpx control or G12D_1–23_ peptide–Lpx. Significance was determined using a one-sided Student’s *t-*test (**b**–**d**). (*p < 0.05. Error bar = mean ± s.e.m.).

T cell recognition of tumor cells requires peptide-MHC presentation. The level of MHC expression may dictate whether the vaccine-activated T cells can impact tumor growth. Although high levels of MHC class I were expressed in vitro before implantation (Fig. 6a), MC38-G12D cells significantly downregulated MHC class I in vivo (Fig. 6b). However, after immunization, we observed increased tumor-specific MHC class I and II expression, indicating that cationic lipoplex vaccines containing neoantigen-specific peptides may also sensitize the tumor microenvironment due to antigen recognition by TILs (Fig. 6b and Supplementary Fig 8a). Moreover, this upregulation of MHC was restricted to tumor cells as MHC class I and II levels on tumor-infiltrating myeloid cells were not altered after immunization (Fig. 6c and Supplementary Fig 8a). In line with further activation and potential recognition of tumor cells by the immune system, PD-L1 expression on the tumor cells was increased after immunization (Fig. 6d and Supplementary Fig 8b). These data suggest that the effector response elicited by the peptide–lipoplex vaccine may be impeded by tumor-specific expression of PD-L1.

### Checkpoint inhibition in combination with peptide–lipoplex vaccination enhances vaccine responses

**Fig. 7:**
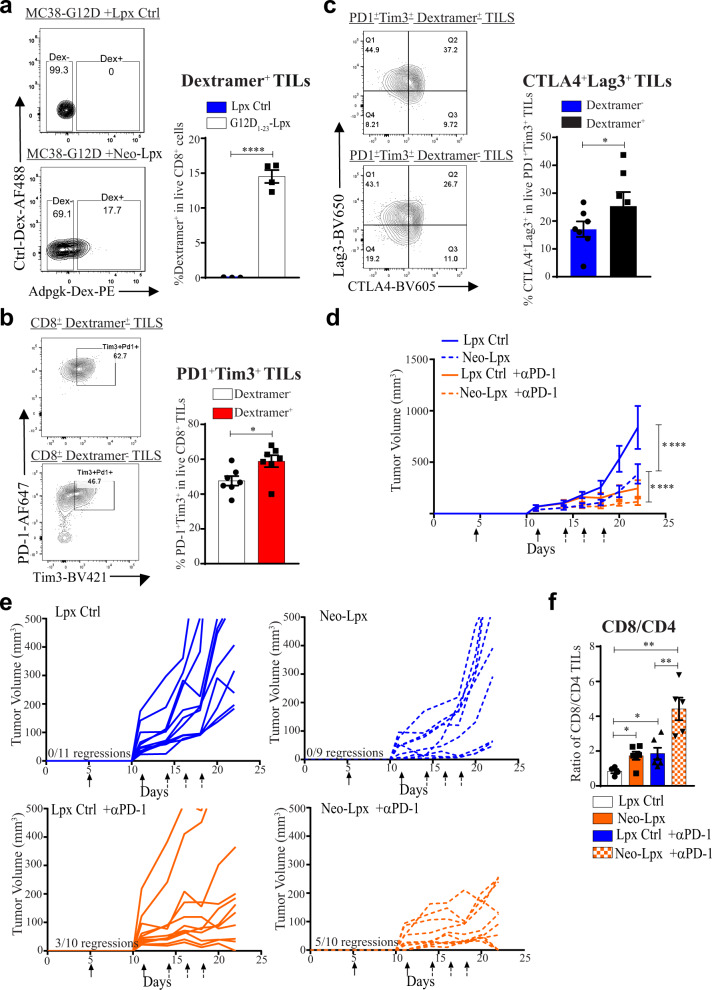
Therapeutic peptide–lipoplex vaccination in combination with checkpoint inhibition elicits potent anti-tumor responses with exhausted TILs. **a** Detection of mutant Adpgk-specific CD8^+^ TILs in mice immunized with Neo-Lpx using an MHC class I dextramer specific for Adgk. **b**, **c** TILs specific or not for Adpgk using dextramer staining were analyzed for PD-1 and Tim3 expression (**b**) and CTLA4 and Lag3 expression (**c**). **d** Mutant Adpgk and Copg1 27-mers with G12D_1–23_ peptide were formulated lipoplexes (Neo-Lpx) to immunize mice with MC38-G12D tumors 5 days after implantation (solid arrows). Mice were immunized and/or treated with PD-1 antibody or isotype control after randomization on day 14 (dashed arrows). **e** Tumor growth curves of individual mice from (**d**). Significance was determined using two-way ANOVA (****p < 0.0001, ***p < 0.001, *p < 0.05. Error bar = mean ± s.e.m. *n* = 9–10). **f** Tumor-infiltrating CD4^+^ and CD8^+^ T cells from the different mouse groups taken down on day 23 after MC38 tumor implantation. Significance was determined using two-way ANOVA and one-sided or paired two-tailed Student’s *t*-test (**b**, **c**, **f**). (*p < 0.05, **p < 0.01. Error bar = mean ± s.e.m. *n* = 3–6).

We confirmed the antigen-specific effect of the vaccine by analyzing TILs on days 23–26 and staining with MHC class I dextramers that detect Adpgk-reactive T cells. We found that CD8^+^ TILs specific for Adpgk were enriched in tumors after Neo-Lpx treatment (Fig. 7a). Due to heterogeneity in antigen specificity and activity of TILs, it was important to analyze TILs that respond to the tumor antigens. Thus, we focused on markers of chronic antigen stimulation that may lead to functional T cell exhaustion. We observed high levels of PD-1 and Tim3 on all the TILs. Using the Adpgk dextramers, we compared the lipoplex vaccine-expanded TILs to bulk TILs and found that the vaccine-expanded TILs have a higher dysfunctional signature, as measured by PD-1, Tim3, Lag3, and CTLA4 expression (Fig. 7b, c).

PD-1 axis checkpoint inhibitors have been approved for the treatment of many different types of cancer and higher intratumoral PD-L1 levels can predict response. Because of the higher expression of PD-L1 on tumors and PD-1 on antigen-specific T cells after Neo-Lpx vaccination, we examined whether combining anti-PD-1 with the lipoplex vaccine approach might further block tumor growth. Tumor-bearing mice were vaccinated with the Neo-Lpx vaccine on day 5 after implantation and further randomized at day 14 based on equivalent tumor dimensions and either administered anti-PD-1 or an isotype control antibody as indicated. In these experiments, the combination of Neo-Lpx and checkpoint inhibitor blocked tumor progression much more profoundly than in the isotype control-treated or anti-PD-1-treated empty Lpx groups and resulted in partial or complete responses in 6 of 10 animals (Fig. 7d, e). Consistent with the reduction in tumor size, the tumors that did not shrink in response to anti-PD-1, Neo-Lpx, or the combination therapy had a significantly higher number of CD8^+^ TILs compared to CD4+ TILs (Fig. 7f), showing that the monotherapy induction of CD8^+^ TILs is not sufficient for complete tumor clearance or reduction. These data highlight the beneficial effect of combining vaccination against tumor neoantigen with checkpoint inhibitors.

As encouraging as vaccination against tumor neoantigens can be, our data, in addition to the previous studies^5,22^, reveal that functional T cell exhaustion results in decreased vaccine efficacy, and thus it is not surprising that combining the neoantigen vaccine with PD-1 blockade results in enhanced efficacy. However, the expression of other inhibitory receptors makes it difficult to curtail functional T cell exhaustion in CD8^+^ tumor-reactive TILs. These observations suggest that a peptide-containing lipoplex neoantigen vaccine, with as few as three neoantigens represented, in combination with checkpoint inhibition, may be sufficient to treat established tumors.

## Discussion

The aspiration of neoantigen cancer vaccine design is to find approaches capable of eliciting both robust cytotoxic CD8^+^ T cell responses and CD4^+^ T cell help, the key to establishing memory^24,25^. To date, peptide-based vaccination has been underwhelming. The particular concern is that these peptide-based vaccines targeting neoepitopes predicted to be presented by MHC class I alleles have largely yielded class II reactive CD4^+^ cells^5,26,27^. This study provides clear evidence that a vaccine specifically designed to deliver peptides to lymphatic organs can consistently induce both CD4^+^ and CD8^+^ T cell responses that drive tumor regression, particularly when combined with checkpoint inhibition. The appeal of peptide-based cancer vaccines has been their relatively good safety profiles, ease of manufacturing, and quality control. Our targeted delivery approach combines the known safety of peptide vaccination with liposome formulation that proves a superior alternative to conventional immunization strategies.

Peptide vaccination has been extensively used in the recent past with rather limited benefit in cancer patients—an overall objective response rate of 2.9% out of 440 individuals with metastatic cancer^5,6,28^. The advent of personalized tumor neoantigen vaccines has facilitated the selection of a wider variety of tumor-specific antigens. As a result, several studies have successfully used synthetic peptides targeting several tumor-specific neoantigens in melanoma and glioblastoma^5,6,29^. Although the neoantigens were selected based on their likelihood to stimulate CD8^+^ T cells, CD4^+^ T cells were the dominant population responding to the vaccines, lending credence to the essential role of activating CD4^+^ T cells for the development of a promising cancer vaccine, but also raising questions with regard to the efficiency of cross-presentation that results from peptide vaccination.

Novel platforms are being developed to target the neoantigen vaccines to lymphoid tissue-specific dendritic cells. Kranz et al.^17^ demonstrated the use of cationic particles complexed to mRNA to deliver antigens to DCs and initiate potent anti-tumor responses. In a different study, the same group utilized naked mRNA administered percutaneously into inguinal lymph nodes that led to sustained progression-free survival^26^. We explored the possibility of using similar cationic liposomes to deliver synthetic peptides and found similar biodistribution to the spleen and increased anti-tumor responses in our study.

Other vaccines using *Listeria monocytogenes* as a platform take advantage of the fact that this attenuated bacterium is phagocytosed by dendritic cells and can, when altered, cross-present and activate both CD4^+^ and CD8^+^ T cells^30^. Preclinical data showed some efficacy in a preventative setting when using KRAS-G12D as the only neoantigen^31^, but the challenges of personalized *Listeria* vaccine production must be overcome, given the lengthy manufacturing process and likely regulatory hurdles of delivering live bacteria to patients. Recently, a melittin-based lipid nanoparticle vaccine targeted the lymph node due to its size and particle charge^32^ and stimulated both CD4^+^ and CD8^+^ T cells by lysing tumor cells and activating myeloid cells tumor cell lysis and myeloid cell activation. Similar to this study, we believe that direct targeting of antigens to myeloid cells can increase the propensity for MHC class I- and class II-restricted epitopes to be presented. In an elegant study using lipoprotein nanodiscs, Moon et al.^33^ improved delivery of the antigens to lymphoid organs, leading to potent CD4^+^ and CD8^+^ T cell responses that, when combined with checkpoint inhibition, led to profound elimination of tumors in mice. However, although the responses to the MC38 antigen Adpgk were significantly elevated, the CD8^+^ T cells were driving the responses. In our platform, we observed skewing of CD4^+^ T cell responses when using the Adpgk 27-mer.

Using liposomes to deliver cargo has been investigated for many decades without much success. Interestingly, early studies focused on delivering drugs to the tumor and disregarded the propensity of cationic liposomes to migrate to the spleen and liver^34^. One way to improve the circulation half-life and targeting to cells in the lymphoid tissues is by subcutaneous administration of the cationic liposome-peptide complexes adjacent to lymph nodes. In this scenario, splenic myeloid cells internalize the peptides by macropinocytosis, leading to CD4^+^ and CD8^+^ T cell priming^35^.

Our study used the recurrent putative neoantigen KRAS, which has been shown previously to elicit both CD4^+^ and CD8^+^ T cell responses in mice and humans in an MHC allele-dependent manner. A significant portion of tumors harbors mutations in KRAS, with KRAS-G12D being the most common in pancreatic cancers. Although there is substantial debate in the field, several MHC class I alleles have been reported to have a high affinity to the KRAS mutations G12D and G12V^13,14,36–38^. While only a fraction of the common KRAS mutations is predicted to yield high-affinity HLA class I-binding mutant peptides, HLA-A*02:01 and HLA-A*11:01, two of the most frequent HLA alleles in many populations^39^, have been identified as not only binders of short peptides containing the G12D and G12V mutations, but have also been reported to stimulate CD8^+^ T cells^36,37^. In several patients, CD4^+^ T cells have also shown reactivity to KRAS-G12D^14^, illustrating the potential for several epitopes nested within mutant KRAS to stimulate helper and cytotoxic T cells. Combining an approach that would elicit potent CD4^+^ and CD8^+^ T cell responses may result in more durable anti-tumor responses. We have shown that encapsulating peptides in liposomes leads to an enhanced presentation of different regions of the peptide containing the mutation, leading to stimulation of both CD4^+^ and CD8^+^ T cells. The paucity of CD8^+^ T cell responses we observed with “naked” peptide vaccine sheds light on a potential contributory factor to the underwhelming outcomes from previous clinical trials assessing peptide vaccination targeting in KRAS^40–42^.

Taken together, these observations indicate that the neoantigen vaccine alone may be insufficient to induce complete tumor regression, as the resulting enhanced immunogenicity that follows antigen-specific T cell infiltration will upregulate PD-L1. Moreover, post-vaccine administration, the neo-epitope-specific T cell infiltrate was largely PD-1^+^Lag3^+^Tim3^+^, markers associated with T cell dysfunction and exhaustion in infection and cancer^43,44^. Merely targeting a single pathway, such as the PD-1/PD-L1 pathway, may not be sufficient to restore T cell function in all cases^45^.

Hallmarks of effective vaccine immunotherapy will be the requirement to enhance the number and quality of tumor-infiltrating T cells and the induction of robust CD4^+^ and CD8^+^ T cell responses. Combinatorial approaches, such as the use of checkpoint inhibitors, are also likely to be required. Our study suggests that designing peptide–lipoplexes or other platforms, such as neoantigen-encoding RNA modifications^46^, that can generate a large repertoire of T cell responses has the potential to provide several epitopes for diverse MHC class I and II allelotypes. While further refinement is clearly required, the modular nature of generating peptide–lipoplexes with a variety of tumor neoantigens makes this vaccine platform a candidate for further investigation in a translational setting.

## Methods

### Cell lines and mice

Female 8–12-week-old C57Bl/6 (B6) and BALB/c mice were bought from Charles River Laboratories and kept in pathogen-free conditions in accordance with the Institutional Animal Care and Use Committee (IACUC) and Association for Assessment and Accreditation of Laboratory Animal Care (AAALAC) approved policies. Animal procedures were conducted in accordance with guidelines approved by the IACUC and AAALAC. KRAS-LSL/Lkb1^−/−^ were licensed from the Salk Institute. Expression of G12D was tracked by luciferase expression resulting from the combination of a ROSA-lox-stop-lox allele of firefly luciferase as a surrogate marker for transgene expression. Lung tumors were induced by intra-tracheal inhalation of 50 × 10^6^ plaque-forming units adenovirus-Cre (purchased from University of Iowa adenoviral core) as previously described^21^. Mice that displayed clinical signs of disease, such as labored breathing or severe weight loss, were euthanized and necropsied. CT26 and MC38 cell lines were purchased from ATCC. MC38 cell lines overexpressing the KRAS mutations were made by transducing MC38 cells with retrovirus made by transfecting the Eco/Amph packaging cell line gp293 (Takara Bio) with pMIG containing the mutated KRAS protein (G12C, G12D, or G12V). Cells were tested for mycoplasma every 3 months.

### Mouse models

For immunogenicity studies of mutated KRAS peptides, age-matched female C57BL/6 or BALB/c mice were vaccinated on days 0 and 7 with 100 μg of peptide and 50 μg of CpG (ODN 1826 from Invivogen) per mouse. The readout was performed 6–8 days after the last immunization. Vaccination with lipoplexes was performed either by i.v. injection or subcutaneous (s.c.) injection of 50 μg total cationic liposome complexed with 5 μg synthetic long peptide and 2.5 ng CpG formulated in PBS (200 μl per mouse) into the lateral flank. For therapeutic tumor experiments, C57BL/6 mice were inoculated s.c. with 250,000 MC38 cells into the flank and randomly distributed into treatment groups. For the CT26 model, 200,000 tumor cells were implanted subcutaneously in the flank of Balb/C mice. Tumor volume was measured unblinded with a caliper. Tumor growth was recorded as mean tumor size with standard error.

In the KRAS-LSL/Lkb1^−/−^ model, we tracked tumor development by luciferase expression by total flux (photons s^−1^), which is proportional to tumor grade and tumor burden. Regions of interest (ROI) were quantified as average radiance (photons s^−1^ cm^−2^ sr^−1^, represented by color bars). Subcutaneous administration of peptide lipoplexes was started at week 2 after the administration of Ad-Cre and continued every 7 days for three doses. Tumor growth was traced unblinded by bioluminescence imaging after i.p. injection of an aqueous solution of d-luciferin (200 μl, 3 mg/mouse, PerkinElmer) on a Xenogen IVIS-200 (PerkinElmer). Twelve minutes after injection emitted photons were quantified. In vivo bioluminescence in regions of interest (ROI) were quantified as total flux (photons s^−1^) (IVIS Living Image 4.0). In some experiments, repeated doses (200 μg/mouse i.p.) of CD8-depleting (clone 2.43, BioXcell) or CD4-depleting (clone GK1.5, BioXcell), or CD25-depleting (PC61, BioXcell) antibodies were administered every 2–3 days. CD4- and CD8-depleting antibodies were given 2 days prior to immunization and 10 days before tumor implantation. The experimental group sizes were approved by the regulatory authorities for animal welfare after being defined to balance statistical power, feasibility, and ethical aspects.

### Liposomes

Cationic liposomes (positive net charge) were used to make the peptide–liposome complexes. These were made with DOTMA, DOPE, and DOPC (Avanti Polar Lipids). For some experiments to optimize the liposome formulation, the anionic lipid DOPS was used instead of DOTMA. For the biodistribution studies, we used rhodamine-labeled DOPE (Avanti Polar Lipids). Liposomes were produced by the thin-film method. Briefly, chloroform stock solutions of the individual lipids were prepared at a concentration of ~25 mg ml^−1^ and appropriate amounts (volumes) of the stock solutions were mixed according to the intended lipid ratio. For most experiments, we used a molar ratio of 50:30:20 (DOTMA:DOPE:DOPC). The chloroform was evaporated by streaming argon in the solution and the obtained lipid film was dried overnight. The dry film was hydrated with RNase-free water by and mixed with the synthetic long peptide at a ratio of 10:1 (w/w) lipid:peptide. The peptide–liposome solution was incubated with shaking for 1 h to obtain a raw colloid. For size adjustment, the dispersion was then extruded ten times through polycarbonate membranes with 200 nm pore size using a mini-extruder (Avanti Polar Lipids). Liposome size, polydispersity index, and zeta potential (triplicates) were determined by dynamic light scattering using a Zetasizer (Malvern Instruments).

### Peptide–Lpx preparation and immunization

A diversity of formulations complexed with the peptide were assembled, with liposomes comprising different ratios of the peptide and cationic or anionic lipids. Further optimization was done to complex CpG (ODN 1826, Invivogen) to the peptide–liposomes to achieve stability of the lipoplexes (Lpx). For formulation screening studies, 50 μg peptide–Lpx corresponding to 5 μg of peptide per mouse was injected intravenously (i.v.) or subcutaneously (s.c.). For immunological and tumor experiments, mice were immunized two or three times with 50 μg peptide–Lpx-CpG unless stated otherwise. Control mice received empty Lpx (no peptide) or remain unimmunized. Arrows in vaccination schemes indicate immunization.

### Synthetic peptides

The following peptides were synthesized by Bio-Synthesis (Lewisville, TX): KRAS WT (WT-LP): MTEYKLVVVGAGGVGKSALTIQLIQ; KRAS G12C (G12C_1–23_): MTEYKLVVVGACGVGKSALTIQLIQ; KRAS-G12D (G12D_1–23_): MTEYKLVVVGADGVGKSALTIQLIQ; KRAS G12V (G12V_1–23_): MTEYKLVVVGAVGVGKSALTIQLIQ; Adpgk 27-mer: TGIPVHLELASMTNMELMSSIVHQQVF; Copg1 27-mer: DSPLFDFIESCLRNEHEMVVYEAASAI. Adpgk 9-mer: ASMTNMELM and the Copg1 9-mer: SCLRNEHEM; overlapping 15-mer and 9-mer peptide mixes for G12D_1–23_, and Cy5-labeled G12D_1–23_ at the N-terminus. Tmem87a (G63R) 27-mer: QAIVRGCSMPGPWRSGRLLVSRRWSVE, Slc4a3 (T373I) 27-mer: PLLPFYPPDEALEIGLELNSSALPPTE: E2f8 (I522T) 27-mer: VILPQAPSGPSYATYLQPAQAQMLTPP.

### Tissue preparation

Single-cell suspensions of splenocytes were prepared in PBS by mashing tissue against the surface of a 70-μm cell strainer (BD Falcon) using the plunger of a 5-ml syringe (BD Biosciences). Red blood cells were removed by lysis with ACK buffer (Gibco). In some experiments, lymph nodes and spleens were digested with collagenase D (1 mg ml^−1^; Roche) and passed through cell strainers. For in vivo studies, the tumors were harvested, weighed, minced, and dissociated using the tumor dissociation kit (Miltenyi Inc) and the gentleMACS Dissociator (Miltenyi Inc) following manufacturers instructions. Cells were then washed with complete RPMI and filtered to remove clumps. Cells were then surface stained with the indicated antibodies before fixation and permeabilization (if needed for intracellular staining). To culture bone marrow-derived dendritic cells (BMDCs), bone marrow cells were flushed from femurs and tibia bones, homogenized and filtered, before red blood cells were lysed with ACK buffer. BMDCs were made from bone marrow with murine 20 ng ml^−1^ GM-CSF and 5 ng ml^−1^ IL-4 (Peprotech) for 6 days in 37 °C.

### Flow cytometry

Monoclonal antibodies for extracellular staining were purchased from Biolegend or BD Biosciences. These included, CD11b, CD11c, CD4, CD8, CD25, CD44, CD45, Ia/Ie, H2K^b^, H2D^b^, PD-L1, PD-1, CTLA4, and Lag3. Intracellular staining was performed with antibodies against IFN-γ, IL-17, IL-2, Foxp3, and Ki67 using the cytofix/perm kit from eBioscience either with or without stimulation of 2–5 × 10^6^ splenocytes with 10 μg ml^−1^ G12D_1–23_ or irrelevant peptide for 8 h at 37 °C (BD GolgiStop monensin was added for the last 2 h of incubation). Tumor-infiltrating leucocytes were prepared from subcutaneous MC38 tumors (15–20 days after implantation) or lungs from KRAS-G12D GEM model (6–7 weeks post Ad-Cre administration). Tumors were harvested and minced into pieces of 1–2 mm diameter. The resulting cell suspension was harvested, filtered through a 70-mm cell strainer, washed two times, and stained for surface and intracellular markers. Cells were washed with PBS and labeled with Live/Dead fixable Blue dye (Molecular Probes) before adding extracellular staining antibodies. Quantification of Adpgk and Copg1-specific T cells was done with a Adpgk/H2D^b^ and Copg1/H2D^b^ dextramers generated by Immudex.

### Enzyme-linked ImmunoSpot (ELISpot)

After immunization with the peptide or peptide–Lpx splenocytes were cultured for 18 h at 37 °C in anti-IFN-γ-coated Multiscreen 96-well plates from BD Biosciences or Immunospot (dual IFN-γ and ELISpot) following the manufacturer’s instructions. For stimulation, either 10 μg ml^−1^ of the peptide was added to 1 × 10^6^ splenocytes or LPS matured-BMDCs for co-culture with CD4^+^ or CD8^+^ T cells isolated using MACS microbeads for positive or negative selection (Miltenyi). For analysis of tumor-infiltrating lymphocytes, single-cell suspensions from tumors were obtained. CD4^+^ and CD8^+^ TILs were isolated using MACS microbeads (Miltenyi). TILs were co-incubated with T cell-depleted splenocytes for 18 h (ELISpot) or 8 h (FACS in the presence of GolgiStop monensin). All samples were tested in duplicates or triplicates.

### In vitro uptake studies

BMDMs and BMDCs generated as listed above were incubated with Cy5-peptide–Lpx for 10 min at 37 °C and washed thoroughly with PBS to remove extracellular peptide–Lpx. Cells were stained and run on a flow cytometer. For inhibition studies, cells were treated with 10 μM Rottlerin (Sigma) for 1 h or cytochalasin D (Sigma) for 3 h before incubation with Cy5-peptide–Lpx for 10 min. Cells were run on a flow cytometer or imaged using an Operetta CLS (PerkinElmer).

### In silico prediction

The MHC-I binding predictions were made using the Immune Epitope Database (IEDB) analysis resource Consensus tool^47^, which combines predictions from ANN^47,48^, SMM^49^, and comblib^50^. The IEDB recommended prediction method was used. Regions spanning the mutations (10 aa positions upstream and 10 aa positions downstream of the mutation) were input into the tool and broken down into all potential 9-mer, 10-mer, and 11-mer peptides. A percentile rank was generated for each peptide based on how that peptide compares to a library of peptides from the SWISSPROT database. A low percentile rank (e.g., <1%) indicates a strong potential MHC binder.

### Bioluminescence imaging

Uptake of Cy5-labeled peptide or rhodamine-labeled liposomes was evaluated by ex vivo bioluminescence imaging using the Xenogen IVIS Spectrum imaging system (PerkinElmer). Upon organ harvest, tissues were directly subjected to fluorescence measurements for Cy5 or rhodamine. The tissues were identified as the regions of interest (ROI), which were quantified as average radiance (photons s^−1^ cm^−2^ sr^−1^) (IVIS Living Image 4.0).

### Statistical analysis

Data are presented as mean ± standard error of the mean (SEM). Values were analyzed using GraphPad Prism version 7 (GraphPad Softwares Inc.). Tumor efficacy studies were analyzed by repeated-measures ANOVA followed by post hoc Dunnett’s analysis to compare tumor growth inhibition.

### Reporting summary

Further information on research design is available in the Nature Research Reporting Summary linked to this article.

## Supplementary information

Supplementary Figures

Reporting Summary Checklist

## Data Availability

The authors declare that all data supporting the findings of this study are available within the paper and its supplementary information files. Extra data are available from the corresponding author upon request.
