# Supplementary material for: A nanoparticle vaccine that targets neoantigen peptides to lymphoid tissues elicits robust antitumor T cell responses
Source: NPJ Vaccines. 2020 Nov 12;5:106. doi: 10.1038/s41541-020-00253-9 (PMC7661730; doi:10.1038/s41541-020-00253-9)
Supplement: Supplementary file 1 — Supplementary Figures [file 41541_2020_253_MOESM1_ESM.pdf]

a

	2	3	4	5	6	7	8	9	10	11	12	13	14	15	16	17	18	19	20	21	22
**WT**	T	E	Y	K	L	V	V	V	G	A	G	G	V	G	K	S	A	L	T	I	Q
**G12C**	T	E	Y	K	L	V	V	V	G	A	C	G	V	G	K	S	A	L	T	I	Q
**G12D**	T	E	Y	K	L	V	V	V	G	A	D	G	V	G	K	S	A	L	T	I	Q
**G12V**	T	E	Y	K	L	V	V	V	G	A	V	G	V	G	K	S	A	L	T	I	Q

Y K L V V V G A G
K L V V V G A G G
L V V V G A G G V
V V V G A G G V G
V V G A G G V G K
V G A G G V G K S
G A G G V G K S A
A G G V G K S A L
G G V G K S A L T

b

allele	length	peptide	Peptide name	percentile	ann_ic50
HLA-A*11:01	9	VVGAVGVGK	V-p9-5	0.55	65.47
HLA-A*11:01	9	VVGACGVGK	C-p9-5	0.75	134.97
HLA-A*03:01	9	VVGACGVGK	C-p9-5	0.75	221.43
HLA-A*03:01	9	VVGAVGVGK	V-p9-5	0.75	202.62
HLA-A*11:01	9	VVGADGVGK	D-p9-5	0.85	368.19
HLA-A*11:01	10	VVVGAVGVGK	V-p10-6	0.85	137.28
HLA-A*02:03	10	KLVVVGACGV	C-p10-8	0.9	62.04
HLA-A*02:03	10	KLVVVGAVGV	V-p10-8	0.95	62.04
HLA-A*02:06	9	LVVVGACGV	C-p9-7	0.95	18.79
HLA-A*03:01	10	VVVGAVGVGK	V-p10-6	0.95	341.99
HLA-A*68:02	9	LVVVGAVGV	V-p9-7	1	127.41
HLA-A*02:06	9	LVVVGAVGV	V-p9-7	1	24.63

c

CGVGKSALT	$G12C_{12-20}$
ACGVGKSAL	$G12C_{11-19}$
GACGVGKSA	$G12C_{10-18}$
VGACGVGKS	$G12C_{9-17}$
VVGACGVGK	$G12C_{8-16}$
VVVGACGVG	$G12C_{7-15}$
LVVVGACGV	$G12C_{6-14}$
KLVVVGACG	$G12C_{5-13}$
YKLVVVGAC	$G12C_{4-12}$

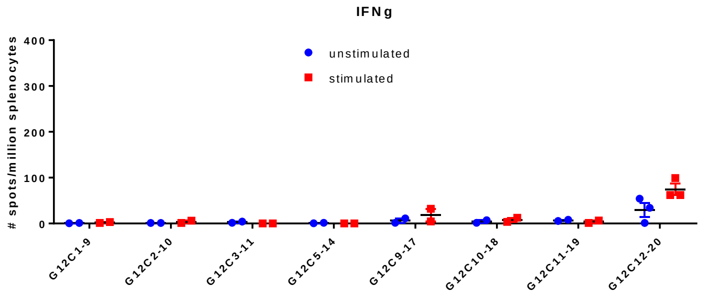

d

DGVGKSALT	$G12D_{12-20}$
ADGVGKSAL	$G12D_{11-19}$
GADGVGKSA	$G12D_{10-18}$
VGADGVGKS	$G12D_{9-17}$
VVGADGVGK	$G12D_{8-16}$
VVVGADGVG	$G12D_{7-15}$
LVVVGADGV	$G12D_{6-14}$
KLVVVGADG	$G12D_{5-13}$
YKLVVVGAD	$G12D_{4-12}$
EYKLVVVGAVGVGKS	$G12V_{3-17}$

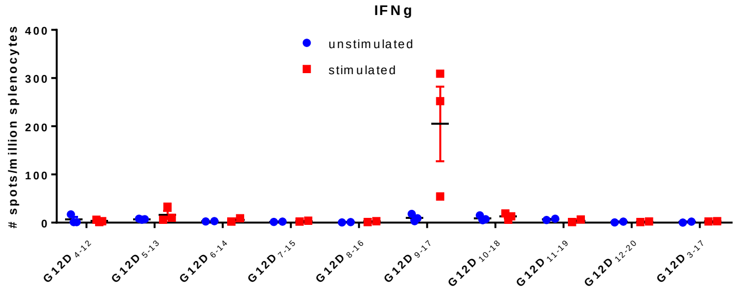

e

VGVGKSALT	$G12V_{12-20}$
AVGVGKSAL	$G12V_{11-19}$
GAVGVGKSA	$G12V_{10-18}$
VGAVGVGKS	$G12V_{9-17}$
VVGAVGVGK	$G12V_{8-16}$
VVVGAVGVG	$G12V_{7-15}$
LVVVGAVGV	$G12V_{6-14}$
KLVVVGAVG	$G12V_{5-13}$
YKLVVVGAV	$G12V_{4-12}$
EYKLVVVGAVGVGKS	$G12V_{3-17}$

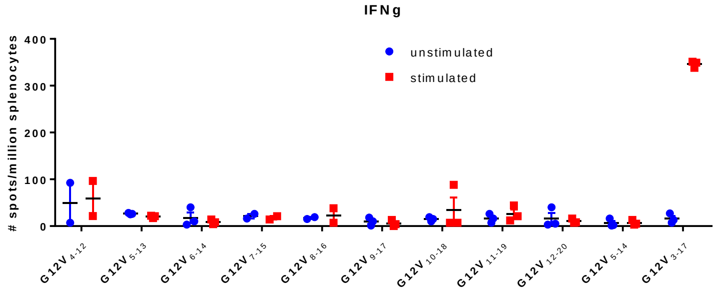

Supplementary Fig. 1 In silico prediction of 9-mer peptides derived from mutant KRAS reveal few potential epitopes in mice. **a** Illustration of 9-mer peptides that are measured according to their potential to bind to different human HLA class I molecules when a 22-mer peptide with the most common KRAs mutations (G12C, G12D, and G12V) is broken up. **b** Predictions of the top binding 9-11-mer peptides with the highest likelihood of binding to different HLA-I alleles determined by the IEDB recommended prediction method. Only weak and strong binders (top 1% rank and IC50 less than 500nM, with lower numbers indicating higher affinity) are shown. **c-e** C57Bl/6 mice were immunized with overlapping 9-mer peptides with the KRAS G12C, G12D, and G12V mutations listed in the table and CpG. After two immunizations 1 week apart, the splenocytes were stimulated with the immunizing peptide in an IFN-γ ELISpot assay.

a

Immunizing peptide G12D$_{1\text{-}23}$: MTEYKLVVVGADGVGKSALTIQL

D1	MTEYKLVVVGADGVG	G12D$_{1\text{-}15}$
D2	TEYKLVVVGADGVGK	G12D$_{2\text{-}16}$
D3	EYKLVVVGADGVGKS	G12D$_{3\text{-}17}$
D4	YKLVVVGADGVGKSA	G12D$_{4\text{-}18}$
D5	KLVVVGADGVGKSAL	G12D$_{8\text{-}19}$
D6	LVVVGADGVGKSALT	G12D$_{6\text{-}19}$
D7	VVVGADGVGKSALTI	G12D$_{7\text{-}20}$
D8	VVGADGVGKSALTIQ	G12D$_{8\text{-}21}$
D9	VGADGVGKSALTIQL	G12D$_{9\text{-}22}$

b

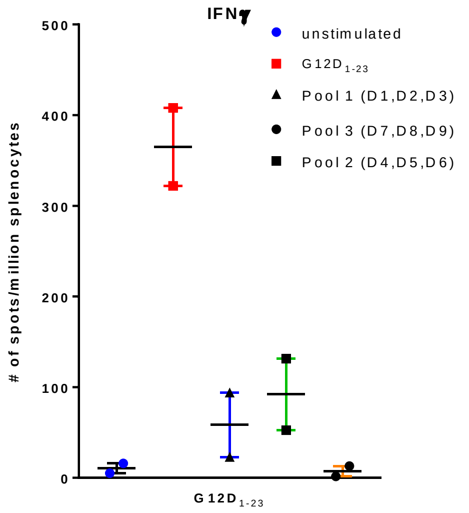

c Immunizing peptides: D4 (G12D$_{9\text{-}17}$) + D5 (G12D$_{8\text{-}16}$) + CpG

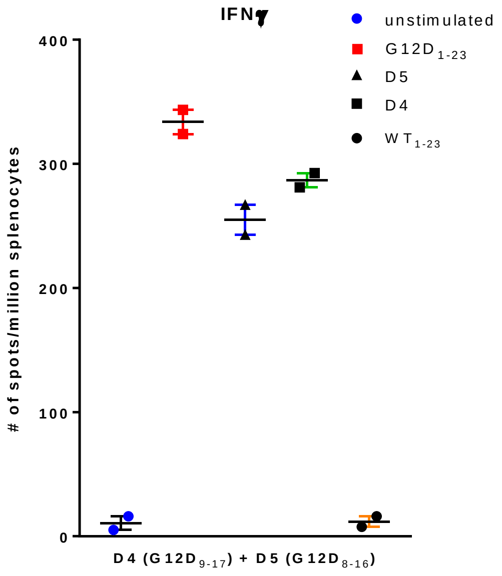

Supplementary Fig. 2. CD4+ T cells respond to 2 different epitopes spanning the KRAS G12D mutation. **a-b** C57Bl/6 mice were immunized with the G12D$_{1\text{-}23}$ 23-mer peptide and CpG twice, one week apart. Splenocytes were stimulated with the G12D$_{1\text{-}23}$ immunizing peptide and 3 pools consisting of 3 overlapping 15-mer peptides with the KRAS G12D mutation listed in the table (n=2). **c** Mice were immunized with D4 and D5 15-mer peptides found to stimulate T cells reactive to G12D$_{1\text{-}23}$ in **b**. After two immunizations 1 week apart, the splenocytes were stimulated with the immunizing peptides, G12D$_{1\text{-}23}$, or WT$_{1\text{-}23}$ in an IFN-γ ELISpot assay.

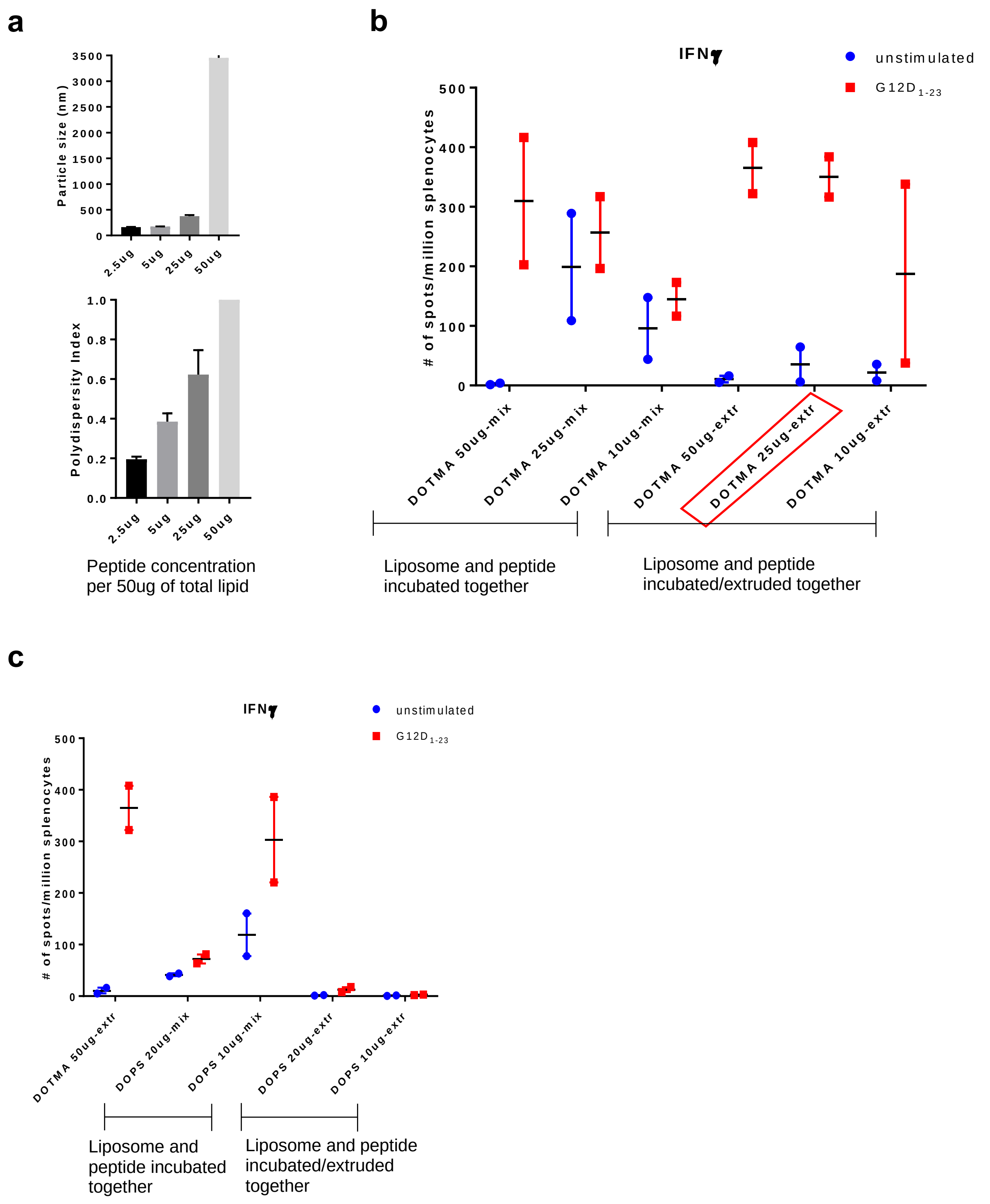

Supplementary Fig. 3. Activation of T cell responses by peptide-lipoplexes is dependent on the concentration and charge of the lipids. **a** Particle size (top) and polydispersity index (bottom) (n=3) of peptide-liposome-CpG (Lpx) generated with $G12D_{1\text{-}23}$ and DOTMA/DOPE/DOPC at various peptide concentrations per 50ug of total liposomes. Zeta potential **b-c** C57Bl/6 mice were s.c. immunized twice with various concentrations of $G12D_{1\text{-}23}$ 23-mer peptide and CpG complexed with different cationic or anionic liposome compositions 1 week apart. Splenocytes were restimulated with the immunizing peptide 1 week after the last immunization (n=2).

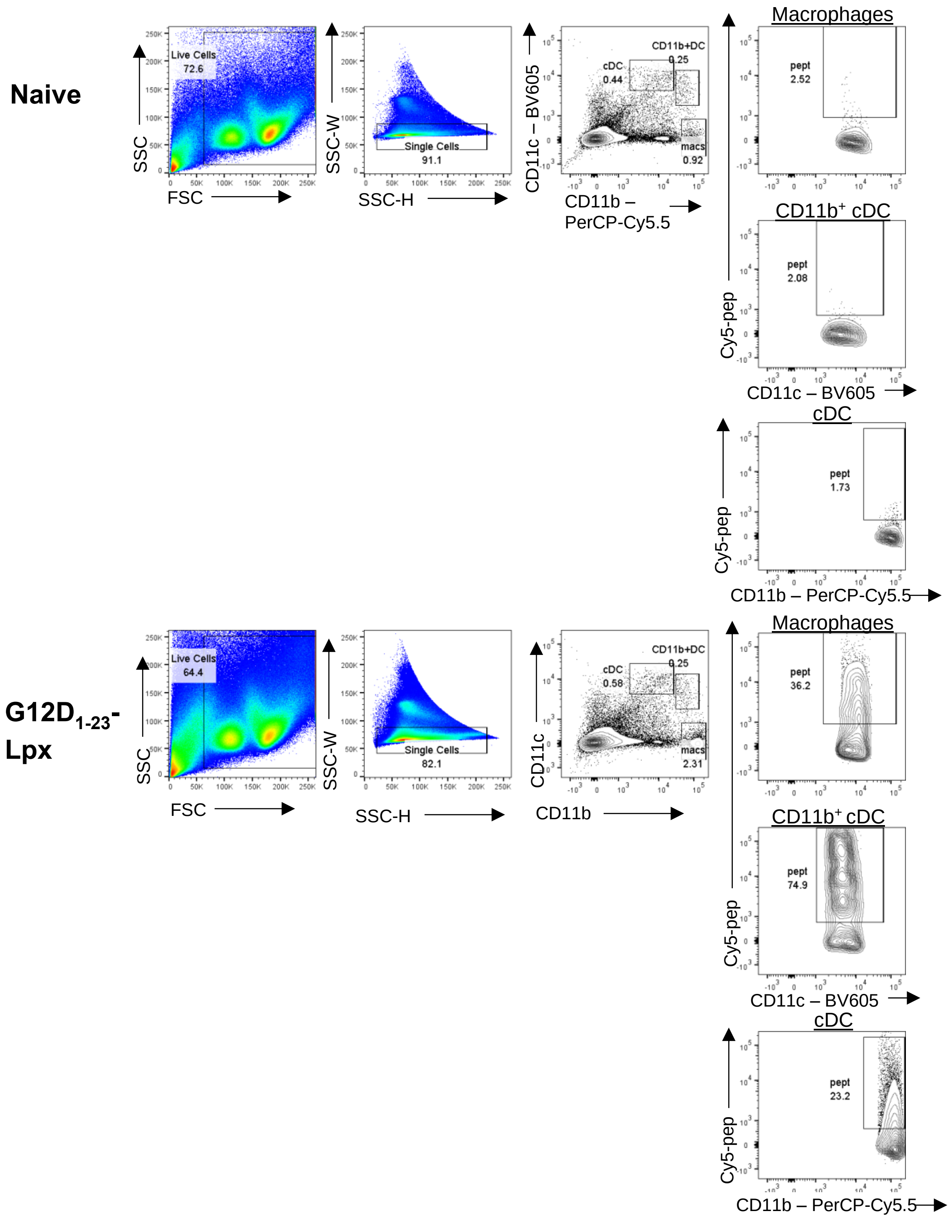

Supplementary Fig. 4. Peptides in lipoplexes are taken up by different populations of myeloid cells. Gating strategy for CD11b+CD11c- macrophages, $CD11b^{int}CD11c^{+}$ cDCs, and $CD11b^{+}CD11c^{+}$ DCs that internalized the Cy5-labeled $G12D_{1\text{-}23}$ peptide 30 min after s.c. immunization of mice with the $G12D_{1\text{-}23}$ peptide-liposome-CpG ($G12D_{1\text{-}23}$-Lpx).

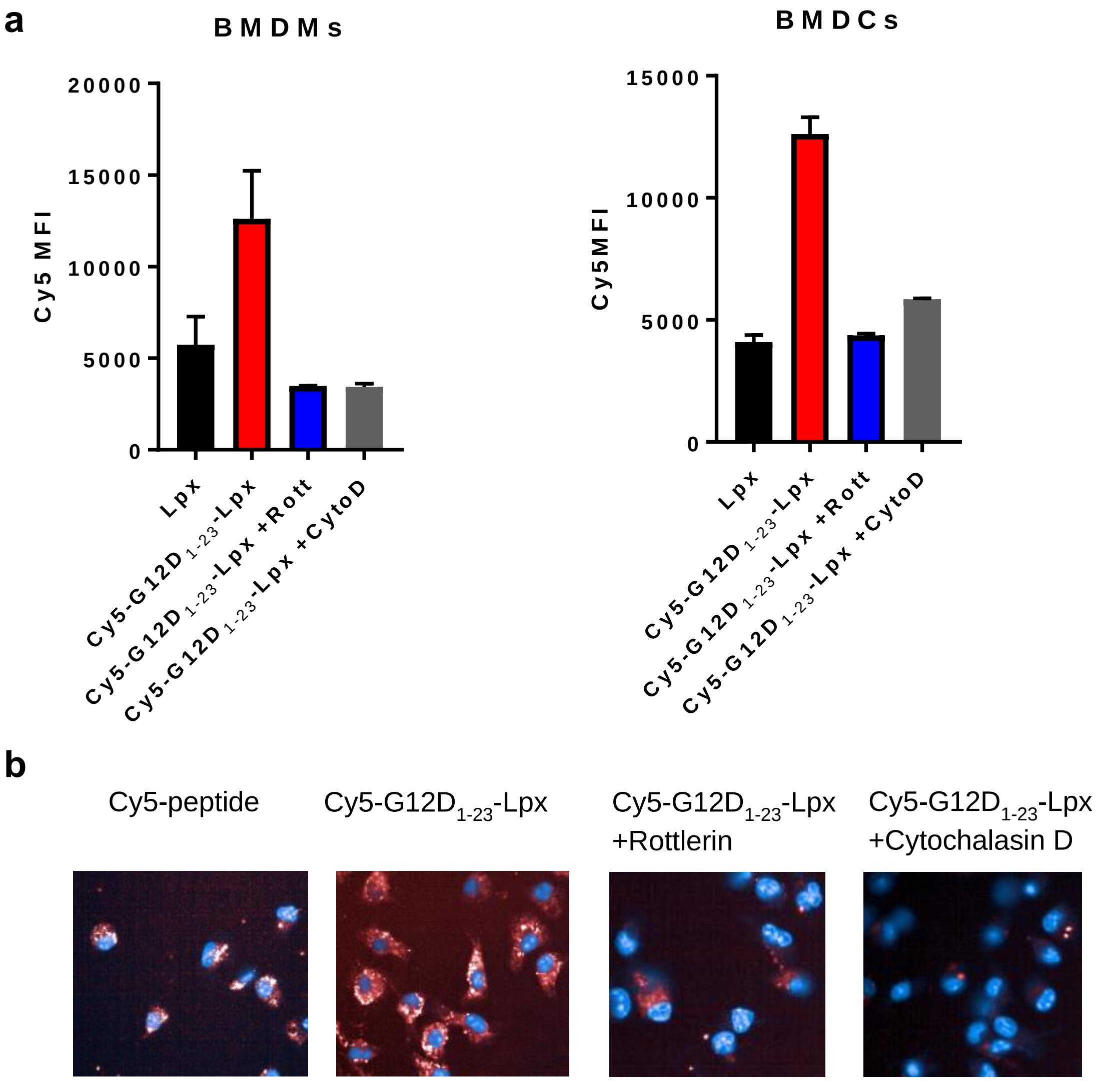

Supplementary Fig. 5. Peptide-lipoplexes are internalized by myeloid cells through macropinocytosis. **a** BMDMs (left) and BMDCs (right) were treated with Rottlerin (Rott) for 1 hr or cytochalasin D (CytoD) for 3 hrs before incubation with Cy5-labeled $G12D_{1\text{-}23}$-Lpx for 10 mins, washed, and run a flow cytometer. **b** Fluorescence imaging of BMDMs incubated with Cy5 peptide, Cy5-peptide-Lpx or the inhibitors Rottlerin and Cytochalasin D. Conditions were run in duplicates (Error bar = mean ± s.e.m.)

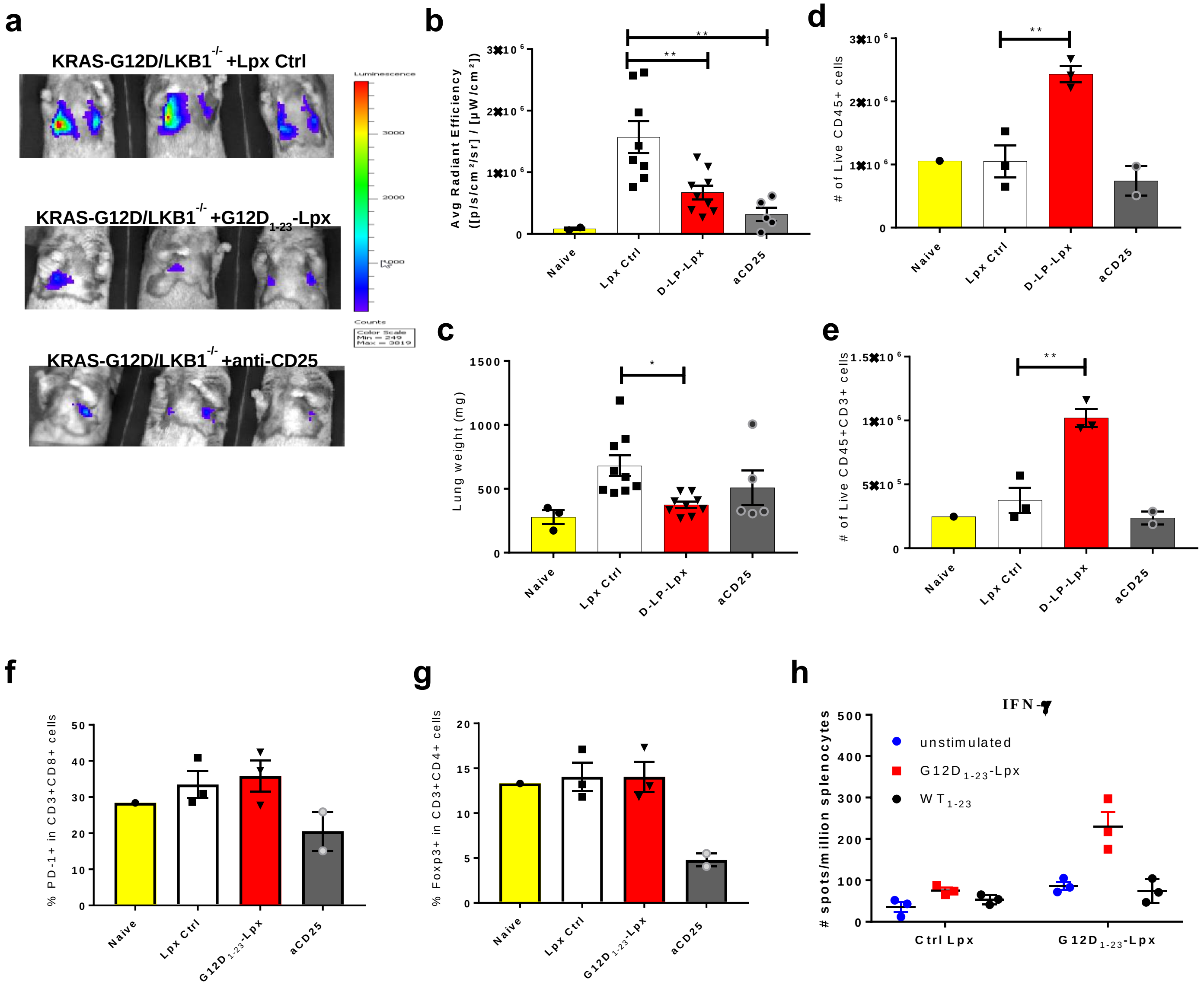

Supplementary Fig. 6. Peptide-lipoplex therapy reduces tumor growth in a KRAS-G12D-driven lung adenocarcinoma model. **a-c** KRAS-G12D/LKB1$^{-/-}$ mice given Cre-expressing adenovirus and immunized with G12D$_{1-23}$-Lpx weekly for 3 doses. Bioluminescence imaging and lung weights after 6 weeks shows beneficial effect of the peptide-Lpx vaccine (n=8-10). **d-g** Total numbers of immune infiltrates (CD45+), T cells (CD3+), CD8+PD-1+ T cells, and CD4+ Foxp3+ regulatory T cells in the lungs of diseased mice (n=2-5) and a naïve mouse as comparison (n=1). **h** KRAS G12D$_{1-23}$ peptide-specific T cells were present in mice immunized with peptide-Lpx vaccine 6 weeks after tumor initiation, as shown by IFN-γ ELISpot. Significance was determined using two-way ANOVA **b,c** and paired two-tailed Student's *t* test **d,e.f** (***p < 0.001, **p <0.01, *p <0.05. Error bar = mean ± s.e.m.).

Supplementary Table 1. MHC class I prediction of MC38- and CT26-specific potential neoantigens.

C57Bl/5 - MC38	Mutated Sequence used for vaccination	Reactive T cell subtype with naked peptide	Reactive T cell subtype with peptide-Lpx	Peptide rank for mutated 9-mer peptide
Adpgk	TGIPVHLELASMTNMELMSSIVHQQVF	CD4+	CD4+ and CD8+	0.01
Copg1	DSPLFDFIESCLRNEHEMVVYEAASAI	CD4+ and CD8+	CD8+	0.08
Kras-G12D	MTEYKLVVVGADGVGKSALTIQLIQ	CD4+	CD4+ and CD8+	18.3

Balb/c - CT26	Mutated Sequence used for vaccination	Reactive T cell subtype with naked peptide	Reactive T cell subtype with peptide-Lpx	Peptide rank for mutated 9-mer peptide
Slc4a13	PLLPFYPPDEALEIGLELNSSALPPTE	CD4+	CD4+ and CD8+	0.78
Tmem87a	QAIVRGCSMPGPWRSGRLLVSRRWSVE	CD4+	CD4+ and CD8+	0.07
E2f8	VILPQAPSGPSYATYLQPAQAQMLTPP	CD4+	CD4+	0.4
Kras-G12D	MTEYKLVVVGADGVGKSALTIQLIQ	CD4+	CD8+	0.95

Predictions determined by IEDB recommended prediction tool, interrogating 9-10-mer peptides from the 27-mer sequence shown.

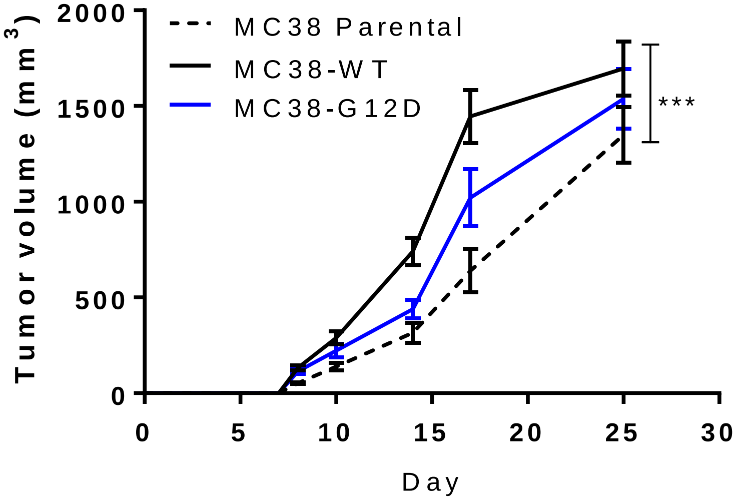

Supplementary Fig 7. MC38-KRAS-WT and MC38-KRAS-G12D tumors grow slightly faster compared to parental MC38 tumors. MC38 cells transduced with retrovirus-expressing KRAS-WT and KRAS-G12D were implanted into C57BL/6 mice and the growth rate was monitored over 25 days. Significance was determined using two-way ANOVA (***p <0.001. Error bar = mean ± s.e.m. n=8-10).

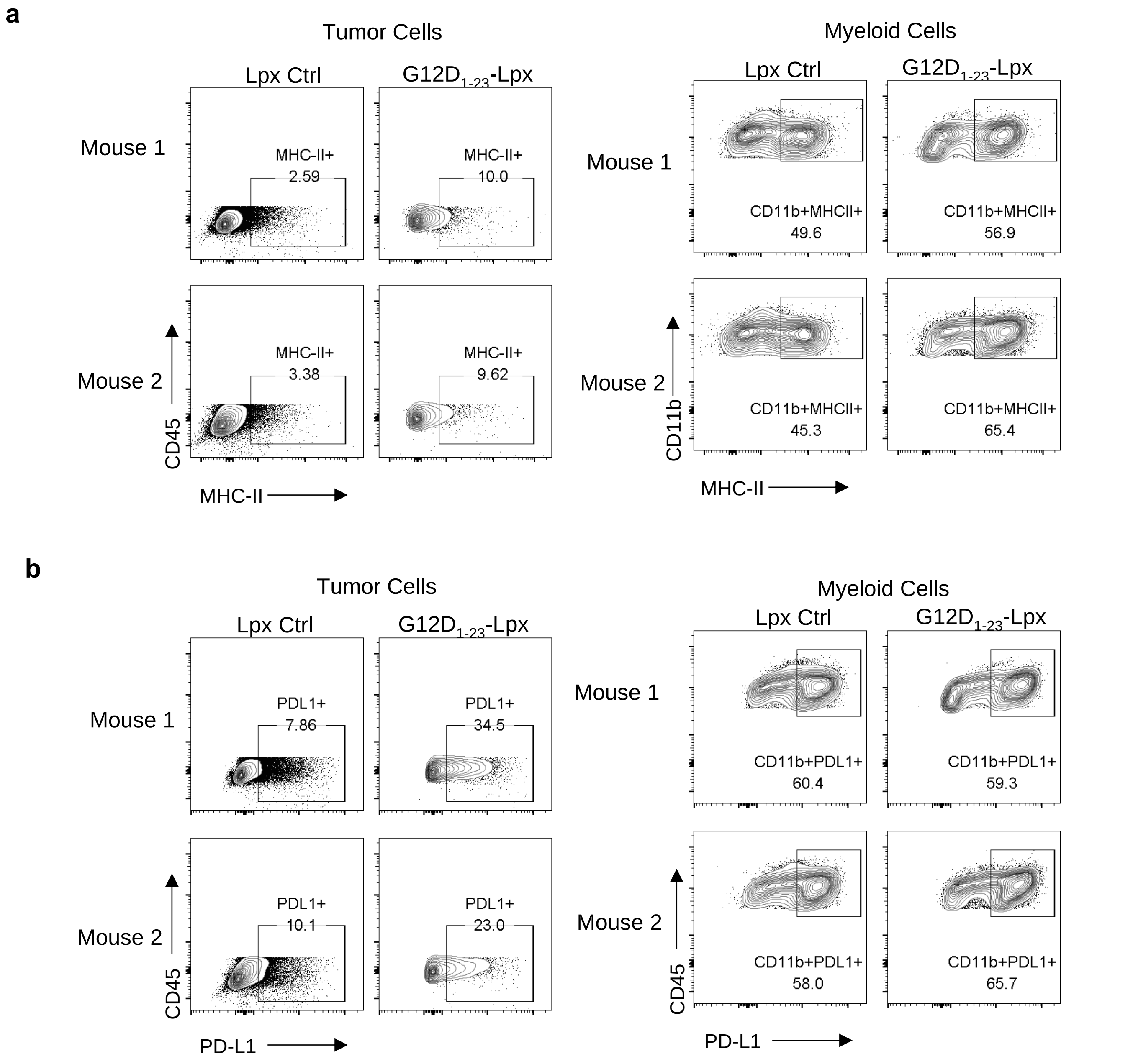

Supplementary Fig 8. Tumors respond to peptide-lipoplex vaccine by upregulating MHC class II and PD-L1. Representative flow cytometric analysis from 2 mice with MC38-G12D tumors treated with Lpx cotrol or the G12D$_{1-23}$-Lpx. **a** MHC class II expression on MC38-G12D tumors (left) or CD11b+ myeloid cells (right) from immunized mice. **b** PD-L1 expression analyzed by flow cytometric analysis on MC38-G12D tumors (left) and tumor-infiltrating myeloid cells (right) in mice immunized with Lpx control or G12D$_{1-23}$ peptide-Lpx.
