# Supplementary material for: A nanoparticle vaccine that targets neoantigen peptides to lymphoid tissues elicits robust antitumor T cell responses
Source: NPJ Vaccines. 2020 Nov 12;5:106. doi: 10.1038/s41541-020-00253-9 (PMC7661730; doi:10.1038/s41541-020-00253-9)
Supplement: Supplementary file 2 — Reporting Summary Checklist [file 41541_2020_253_MOESM2_ESM.pdf]

nature research

Corresponding author(s): James J. Johnston

Last updated by author(s): Sep 28, 2020

Reporting Summary

Nature Research wishes to improve the reproducibility of the work that we publish. This form provides structure for consistency and transparency in reporting. For further information on Nature Research policies, see our Editorial Policies and the Editorial Policy Checklist.

Statistics

For all statistical analyses, confirm that the following items are present in the figure legend, table legend, main text, or Methods section.

n/a	Confirmed	
☐	☒	The exact sample size (*n*) for each experimental group/condition, given as a discrete number and unit of measurement
☐	☒	A statement on whether measurements were taken from distinct samples or whether the same sample was measured repeatedly
☐	☒	The statistical test(s) used AND whether they are one- or two-sided<br>*Only common tests should be described solely by name; describe more complex techniques in the Methods section.*
☒	☐	A description of all covariates tested
☐	☒	A description of any assumptions or corrections, such as tests of normality and adjustment for multiple comparisons
☐	☒	A full description of the statistical parameters including central tendency (e.g. means) or other basic estimates (e.g. regression coefficient) AND variation (e.g. standard deviation) or associated estimates of uncertainty (e.g. confidence intervals)
☐	☒	For null hypothesis testing, the test statistic (e.g. F, t, r) with confidence intervals, effect sizes, degrees of freedom and P value noted<br>*Give P values as exact values whenever suitable.*
☒	☐	For Bayesian analysis, information on the choice of priors and Markov chain Monte Carlo settings
☒	☐	For hierarchical and complex designs, identification of the appropriate level for tests and full reporting of outcomes
☒	☐	Estimates of effect sizes (e.g. Cohen's *d*, Pearson's *r*), indicating how they were calculated

Our web collection on statistics for biologists contains articles on many of the points above.

Software and code

Policy information about availability of computer code

Data collection	Microsoft Excel for Office 365 (in vivo studies, ELISpot, flow cytometry)<br>BD FACSDiva v8.0.1 (flow cytometry)<br>ImmunoSpot v2.6.1 (ELISpot)<br>Living Image v4.5.5 (Imaging)
Data analysis	GraphPad Prism version v7.04 (flow cytometry, ELISpot, in vivo efficacy)<br>FlowJo v10.3 (flow cytometry)<br>Living Image v4.5.5 (Imaging)

For manuscripts utilizing custom algorithms or software that are central to the research but not yet described in published literature, software must be made available to editors and reviewers. We strongly encourage code deposition in a community repository (e.g. GitHub). See the Nature Research guidelines for submitting code & software for further information.

Data

Policy information about availability of data

All manuscripts must include a data availability statement. This statement should provide the following information, where applicable:

- Accession codes, unique identifiers, or web links for publicly available datasets
- A list of figures that have associated raw data
- A description of any restrictions on data availability

The authors declare that all data supporting the findings of this study are available within the paper and its supplementary information files. Extra data are available from the corresponding author upon request.

Field-specific reporting

Please select the one below that is the best fit for your research. If you are not sure, read the appropriate sections before making your selection.

☒ Life sciences ☐ Behavioural & social sciences ☐ Ecological, evolutionary & environmental sciences

For a reference copy of the document with all sections, see nature.com/documents/nr-reporting-summary-flat.pdf

Life sciences study design

All studies must disclose on these points even when the disclosure is negative.

Sample size	Sample size for the peptide screens (n=2-5 per group) with naked peptide or peptide-Lpx was determined using a power calculation with a dichotomous endpoint, comparing two independent study groups (clincalc.com). For tumor efficacy studies (n=7-11), we performed a power calculation such that a minimum change of 32-49% could be detected on the observed data scale. For all calculations, we used alpha 0.05 and power level of 80% using clincalc.com.
Data exclusions	No data were excluded from the studies
Replication	All replication studies were successful, and the detailed information was provided in corresponding figures. In vivo peptide screen studies were performed 2-3 times. All in vivo studies, with the exception of the biodistribution imaging studies and the anti-PD-1 combination studies which were done once, were performed at least 2 times.<br>Independent repeats and samples sizes, as well as statistical analyses and significance levels, are also indicated in the Figure legends or in the Statistics section.
Randomization	When the tumors reached the desired volume (50-100mm^3), mice were randomized into control and treatment groups receiving the vehicle, empty lipoplex controls or the indicated SLP formulation such that each group had a similar mean and SEM before treatment.
Blinding	Data collection and analysis were performed blindly by mixing groups of mice in the same cage.

Reporting for specific materials, systems and methods

We require information from authors about some types of materials, experimental systems and methods used in many studies. Here, indicate whether each material, system or method listed is relevant to your study. If you are not sure if a list item applies to your research, read the appropriate section before selecting a response.

Materials & experimental systems

n/a	Involved in the study	
☐	☒	Antibodies
☐	☒	Eukaryotic cell lines
☒	☐	Palaeontology and archaeology
☐	☒	Animals and other organisms
☒	☐	Human research participants
☒	☐	Clinical data
☒	☐	Dual use research of concern

Methods

n/a	Involved in the study	
☒	☐	ChIP-seq
☐	☒	Flow cytometry
☒	☐	MRI-based neuroimaging

Antibodies

Antibodies used	All antibodies were used at 1:200 unless otherwise noted.<br>anti-mouse CD11b (PerCP-Cy5.5, #45-0112-82, ThermoFisher, Clone M1/70) 1:400<br>anti-mouse CD11c (BV605, #117334, Biolegend, Clone N418) 1:400<br>anti-mouse CD3 (FITC, #100204, Biolegend, Clone 17A2)<br>anti-mouse CD4 (BUV737, #741704, BD Biosciences, Clone RM4-4)<br>anti-mouse CD4 (PerCP-Cy5.5, #100434, Biolegend, Clone GK1.5)<br>anti-mouse CD8 (AF700, #100730, Biolegend, Clone 53-6.7)<br>anti-mouse CD25 (BV711, #102049, Biolegend, Clone PC61)<br>anti-mouse CD44 (APC-Cy7, #103028, Biolegend, Clone IM7)<br>anti-mouse CD45 (APC-Cy7, #557659, BD Biosciences, Clone 30-F11)<br>anti-mouse LAG3 (BV650, #125227, Biolegend, Clone C9B7W)<br>anti-mouse TIGIT (PE-Cy7, #25-9501-82, ThermoFisher, Clone GIGD7) 1:100<br>anti-mouse CD107a (PE, #558661, BD, Clone 1D4B) 1:100<br>anti-mouse PD-1 (AF647, #135230, Biolegend, Clone 29F.1A12)<br>anti-mouse CD28 (BV786 ,#740859, BD, Clone 37.51) 1:100<br>anti-mouse Foxp3 (eF450, #48-5773-82, ThermoFisher, Clone FJK-16s)<br>anti-mouse Ki67 (BV605, #652413, Biolegend, Clone 16A8)

anti-mouse CD11b (PE-Cy7, #101216, Biolegend, Clone M1/70) 1:400
anti-mouse B220 (PerCP-Cy5.5, #103236, Biolegend, Clone RA36B2)
anti-mouse CD3 (BV421, #100336, Biolegend, Clone 145-2C11)
anti-mouse CD45 (APC, #147708, Biolegend, Clone I3/2.3)
anti-mouse IFNg (PE-Cy7, #25-7311-82, eBioscience, Clone XMG1.2)
anti-mouse IL-2 (eFluor450, #48-7021-82, ThermoFisher, Clone JED6-5H4) 1:100
anti-mouse H-2Db (AF647, #111512, Biolegend, Clone KH95)
anti-mouse I-A/I-E (AF700, #107622, Biolegend, Clone M5/114.15.2)
anti-mouse CD274 (PD-L1) (PE, 124308, Biolegend, Clone 10F.9G2) 1:100
anti-mouse PD-1 (PE-CF594, 566833, Biolegend, Clone RMP1-30
anti-mouse Ki-67 (BV605, 652413, Biolegend, Clone 16A8)
anti-mouse Tim3 (BV421, 134013, Biolegend, Clone 134019)
anti-mouse CTLA-4 (BV606, 106323, Biolegend, Clone UC10-4B9)
anti-mouse Lag3 (BV650, 125227, Biolegend, Clone C9B7W)
anti-mouse CD16/32 (#553142, BD Biosciences, Clone 2.4G2)

anti-mouse IFN-γ ELISPOT Kit (BD, 551076)
IFNg/IL2 Double Color ELISpot (ImmunoSpot, mIFNgIL2-1M/10)

Validation

All antibodies were validated by the manufacturers and examples of data are provided on the manufacturers' websites.

Eukaryotic cell lines

Policy information about cell lines

Cell line source(s)

CT26: obtained from the Amgen internal cell bank originally sourced from ATCC
MC38: obtained from the Amgen internal cell bank, originally sourced from the NCI/NIH. The MC38-KRAS-G12D and MC38-KRAS-WT lines were generated by retrovirus transduction with KRAS-G12D or KRAS.

Authentication

Cell lines were not authenticated

Mycoplasma contamination

All cell lines tested negative for mycoplasma contamination.

Commonly misidentified lines
(See ICLAC register)

No commonly misidentified cell lines were used.

Animals and other organisms

Policy information about studies involving animals; ARRIVE guidelines recommended for reporting animal research

Laboratory animals

C57BL/6 or BALB/c mice, 6-12 weeks of age, all female.

Wild animals

The study did not involve wild animals

Field-collected samples

No field-collected samples were involved

Ethics oversight

All studies were performed in accordance with the Institutional Animal Care and Use Committee (IACUC) of Amgen Inc. and the Association for Assessment and Accreditation of Laboratory Animal Care (AAALAC)

Note that full information on the approval of the study protocol must also be provided in the manuscript.

Flow Cytometry

Plots

Confirm that:

☒ The axis labels state the marker and fluorochrome used (e.g. CD4-FITC).

☒ The axis scales are clearly visible. Include numbers along axes only for bottom left plot of group (a 'group' is an analysis of identical markers).

☒ All plots are contour plots with outliers or pseudocolor plots.

☒ A numerical value for number of cells or percentage (with statistics) is provided.

Methodology

Sample preparation

Information provided in the methods section. Briefly, for in vitro studies, cells were enzymatically (Trypsin-EDTA) or non-enzymatically detached from the wells, washed with stain buffer (PBS/FBS) and then incubated with the antibodies. For intracellular cytokine staining, cells were stained with surface antibodies (CD4, CD8, CD3, PD-1, CTLA4, Lag3, CD11b, CD45, etc) before fixation with the eBiosciences Fix/perm buffer set. Cells were then stained with antibodies against IFNg, IL-2, and Foxp3). For in vivo studies, the tumors were harvested, weighed, minced, and dissociated using the tumor dissociation kit (Miltenyi Inc) and the gentleMACS Dissociator (Miltenyi Inc) following manufacturers instructions. Cells were then washed

with complete RPMI and filtered to remove clumps. Cells were then surface stained with the indicated antibodies before fixation and permeabilization (if needed for intracellular staining).

Instrument	BD LSR-II and BD Fortessa analyzers
Software	All analyses were performeded with either BD FACSDiva or FlowJo Software v10
Cell population abundance	The abundance of the relevant cell populations was determined by testing the sorted cells by FACS, with >99% purity.
Gating strategy	The FSC/SSC gate was used to identify lymphocytes and/or tumor cells, followed by exclusion of doublets using SSC-H/SSC-W. Live cells were then gated using the Live/Dead Blue Viability dye. Tumor cells were gated as CD45- cells and immune cells were identified as CD45+ cells. MHC class I and II expression was measured on the CD45- cells. From the CD45+ cells, T cells were CD3+ and CD11b-, whereas myeloid cells were CD11b+ and CD3-. CD8+ and CD4+ T cells were gated independently as single populations. In some cases, the T cells were further gated by CD44 expression, and cytokine expression by IFNg+ or IFNg- was assessed based on cells that were stimulated or not with the immunizing peptide.<br>Gating strategies were shown in Figure 4, Supplementary Figure 4, and Figure 7.

☒ Tick this box to confirm that a figure exemplifying the gating strategy is provided in the Supplementary Information.
